# Bioinspired Brush Reinforced Solid Slippery Coatings for Marine Photovoltaic Protection

**DOI:** 10.1002/advs.202505526

**Published:** 2025-06-10

**Authors:** Ling Yin, Runxiang Tan, Junyi Han, Jianing Wang, Jianjun Cheng, Daheng Wu, Tao Zhang, Liping Wang

**Affiliations:** ^1^ State Key Laboratory of Advanced Marine Materials Ningbo Institute of Materials Technology and Engineering Chinese Academy of Sciences Ningbo 315201 China; ^2^ University of Chinese Academy of Sciences Beijing 100049 China; ^3^ College of Material Science and Engineering Sichuan University Chengdu 610064 China

**Keywords:** bioinspired materials, ion‐dipole interactions, marine photovoltaics, polymer brushes, slippery surfaces

## Abstract

Plant cuticles exhibit exceptional liquid repellence and self‐healing properties through brush‐like cutin‐wax nanostructures, providing inspiration for the multifunctional slippery materials. Here, a plant cuticle‐inspired solid slippery surface (PI‐SSS) is introduced based on surface‐grafted polymer brushes, which act as a stable molecular matrix to enhance the adhesion strength of lubricating copolymer and the substrate (≈0.96 MPa) via strong ion‐dipole interactions. The resultant PI‐SSS demonstrates excellent optical transmittance (≈91.3%) and liquid repellence, particularly against crude oil, alongside multifunctional anti‐biofouling properties (e.g., proteins, chlorella, and mussels). The durability of the coating is validated under extreme conditions, such as prolonged acid and base solution exposure, repeated adhesion/peeling cycles, and seawater immersion, while maintaining its slippery behavior. These features significantly protect solar cells from harsh environments, ensuring a photoelectric conversion efficiency of 15.8% and a stable output voltage of approximately 2.0 V after continuous UV irradiation for a week, and 50 cycles of thermal tests between ‐15 °C and 100 °C, offering a promising approach for marine solar photovoltaic protection.

## Graphical abstract

Plant cuticles exhibit exceptional liquid repellency and self‐healing properties, providing a novel strategy for the multifunctional self‐cleaning materials. A plant‐inspired solid slippery surface (PI‐SSS) based on surface‐grafted poly(ionic liquid) brushes and lubricating copolymer via ion‐dipole interactions are introduced, effectively protecting solar photovoltaics in extreme marine environments.

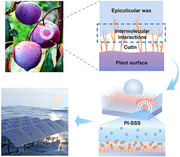

## 1 Introduction

**Figure 1:**
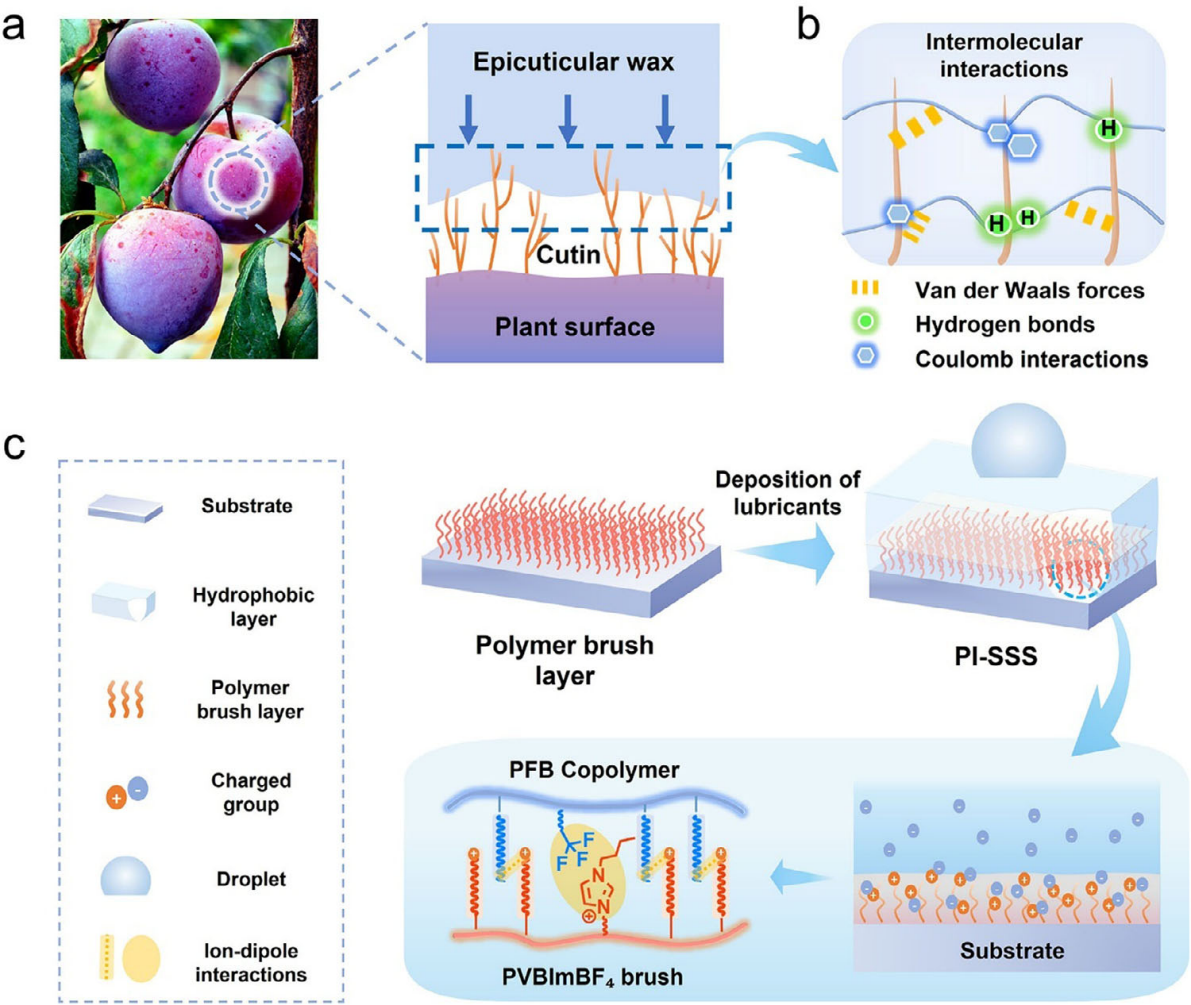
Design principle of PI‐SSS. a) Pictures showing the cuticle of plants, which consists of the epicuticular wax and the cutin. b) Illustration of the intermolecular interaction in the cuticle structure, including van der Waals forces, hydrogen bonds, and Coulomb interactions. c) Fabrication of PI‐SSS via dynamic ion‐dipole interactions and hydrophobic interactions between surface‐grafted polymer chains and lubricating copolymer.

Plant cuticles, the plant‐environment interface covering all aboveground organs (e.g., flowers, stems, fruits, and leaves),^[^ ^1^ ^]^ exemplify nature's blueprint for multifunctional surfaces (**Figure** **1**a).^[^ ^2^ ^]^ These biological barriers exhibit a hierarchical architecture,^[^ ^3^ ^]^ in which functional epicuticular waxes are anchored onto brush‐like cutin network through diverse interactions, including Van der Waals forces, hydrogen bonds and coulomb interactions (Figure 1b),^[^ ^4^ ^]^ resulting in the formation of a slippery^[^ ^5^ ^]^ and anti‐evaporation layer.^[^ ^6^ ^]^ Therefore, the plant surfaces simultaneously achieve self‐cleaning and self‐healing under natural conditions, providing sustained resistance to biofouling (e.g., dust, rain, and pests) by dynamically repelling liquids and contaminants.^[^ ^7^ ^]^

So far, natural synergies have driven the development of bio‐inspired coatings with analogous properties, such as liquid‐infused porous surfaces^[^ ^8^ ^]^ (SLIPSs) and liquid‐like surfaces^[^ ^9^ ^]^ (LLSs), for diverse applications ranging from medical devices to energy systems.^[^ ^10^ ^]^ SLIPSs typically rely on porous substrates infused with lubricating liquids,^[^ ^11^ ^]^ while LLSs are fabricated by grafting flexible polymer brushes onto surfaces to mimic a dynamic and liquid‐like interface.^[^ ^12^ ^]^ However, both systems suffer from poor long‐term stability due to the instability of the lubricant and the easy damage of the ultrathin monolayer, respectively.^[^ ^13^ ^]^ These consequent systems perform effectively in controlled environments, but degrade under mechanical stress, UV radiation, or extreme pH conditions.^[^ ^14^ ^]^ Moreover, integrating optical transparency (>90%) with anti‐biofouling and self‐healing capabilities in a single coating remains unresolved.^[^ ^15^ ^]^ Such fundamental limitations are especially critical for marine solar photovoltaic systems under harsh environments,^[^ ^16^ ^]^ since they fail to provide long‐term protection, resulting in a substantial decrease in energy conversion efficiency by reflecting the light.^[^ ^17^ ^]^

Here, we develop a bioinspired solid slippery surface (PI‐SSS) anchored by surface‐grafted polymer brushes, mimicking the molecular architecture of plant cuticles (Figure 1c). This brush‐like matrix forms a homogeneous scaffold, leveraging strong ion‐dipole interactions to enhance lubricant‐substrate adhesion (≈0.96 MPa), over 300% higher than the blank system (≈0.31 MPa). The PI‐SSS simultaneously achieves high optical transmittance (≈91.3%), universal liquid repellency (including crude oil), and diverse capabilities, such as thermally induced self‐healing, anti‐icing, and resistance to contaminants (proteins, chlorella, and mussels). Importantly, the coating retains its slippery properties under prolonged exposure to seawater, immersion in extreme pH solution, and adhesion/peeling cycles. Further integrated with solar cells, the PI‐SSS enables this panel to maintain a stable voltage output (≈2.0 V) in harsh marine conditions, demonstrating a promising solution for offshore photovoltaic protection.

## 2 Results and Discussion

### 2.1 Preparation and Characterization of PI‐SSS

To mimic the plant cuticle structure, we first prepared the poly(1‐butyl‐3‐vinyliMidazoliuM tetrafluoroborate) (PVBImBF_4_ brushes) (≈99.137 nm) as the cutin structure (Figure 1c) via surface‐initiated copper‐mediated controlled radical polymerization (SI‐Cu°CRP) (Figure S1, Supporting Information).^[^ ^18^ ^]^ Afterwards, (Poly(PFEMA)‐co‐poly(BMA)) (PFB) as a solid lubricant copolymer was coated onto the brushes via a solvent‐free coating method, resulting in the formation of transparent PI‐SSS (Figures S2–S4, Supporting Information) (The specific synthesis process and characteristics are shown in the supporting materials.).^[^ ^19^ ^]^ The infrared spectrum shows a characteristic stretching vibration peak of ‐COO at 1732 cm^−1^ and a characteristic stretching vibration peak of C‐F at 1237 cm^−1^, indicating the successful preparation of PI‐SSS (Figure S5, Supporting Information).^[^ ^20^ ^]^ Scanning electron microscopy (SEM) images and energy dispersive spectroscopy (EDS) images further revealed that the PI‐SSS coating's surface was smooth and dense with a uniform ≈5.925 µm thickness (Figure S6, Supporting Information).

**Figure 2:**
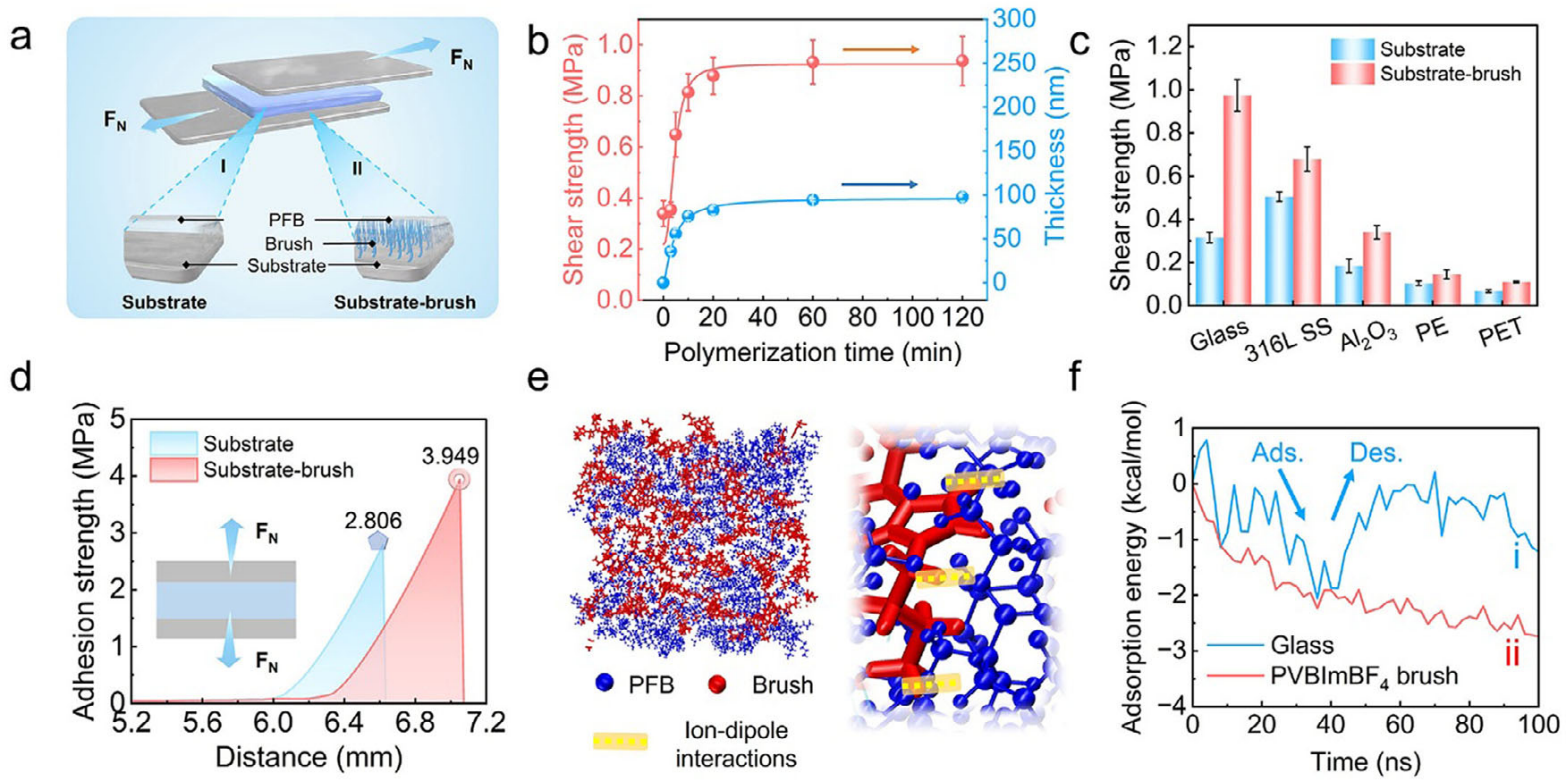
Mechanical properties of PI‐SSS. a) Schematic diagram of shear strength test between PFB coating and substrate. b) Effect of PVBImBF_4_ brushes' thickness on the shear strength of PFB coatings on glass. c) The shear strength of different substrates coated with PI‐SSS (glass, 316L SS, Al_2_O_3_, PE, and PET). d) Adhesion strength of PFB on blank 316 SS and PVBImBF₄ brush coated 316 SS. e) Molecular dynamics snapshot of polymer brushes adhesion with PFB copolymer. f) Variation of the adsorption energy with respect to the time between PFB copolymer with glass substrate and PVBImBF_4_ brushes.

Due to the polar perfluoroalkyl dipoles in the solid PFB copolymer, strong ion‐dipole interactions are established with the imidazolium groups of PVBImBF₄ brushes, enabling the capture of lubricant copolymer and the formation of a stable slippery layer (Figure 1c; Figure S25, Supporting Information).^[^ ^21^ ^]^ The X‐ray photoelectron spectroscopy (XPS) spectrum of F1s is blue‐shifted from 683.6 eV to 686.7 eV, demonstrating the formation of ion‐dipole interactions (Figure S7, Supporting Information).^[^ ^22^ ^]^ Tensile testing indicates that the shear strength of the PI‐SSS coated glass increases from 0.31 MPa to 0.96 MPa (roughly tripling) with increasing brush thickness (**Figure** **2**a,b). Different substrates with growing PVBImBF_4_ brushes, including glass, stainless steel 316L (316L SS), aluminum oxide (Al_2_O_3_), polyethylene (PE), and polyethylene terephthalate (PET), exhibit higher shear strength for the solid fluorinated coating (Figure 2c). Furthermore, the strength required to detach the PFB coating from the polymer brush‐modified surface (3.949 MPa) was significantly higher than that from the blank surface (2.806 MPa) (Figure 2d). These test results indicate that the ion‐dipole interactions can effectively enhance the interfacial adhesion of the solid fluorinated coating to the substrate.

To verify the binding ability of the PVBImBF_4_ brush, molecular dynamics (MD) simulation was further utilized to quantify the interaction between the brush and the blank substrate (SiO_2_) in the presence of PFB copolymer (Figure 2e; Figure S8, Supporting Information).^[^ ^23^ ^]^ Owing to the paucity of interaction sites on the rigid glass substrate, the PFB lubricating copolymer exhibits poor interfacial retention and readily detaches after adsorption (Figure 2f, curve i). In contrast, the polymer brush layer presents a flexible and ionically functionalized interface that enables robust and sustained adsorption via strong ion‐dipole interactions (Figure 2f, curve ii). Meanwhile, density functional theory (DFT) simulations reveal that the binding energy is ‐0.532 eV when the perfluoroalkyl groups interact with the imidazole moieties (Figure S8, Supporting Information).^[^ ^24^ ^]^ These results demonstrate that the ion‐dipole interactions have been successfully established and effectively stabilize the lubricating copolymer.

### 2.2 Slippery Properties

**Figure 3:**
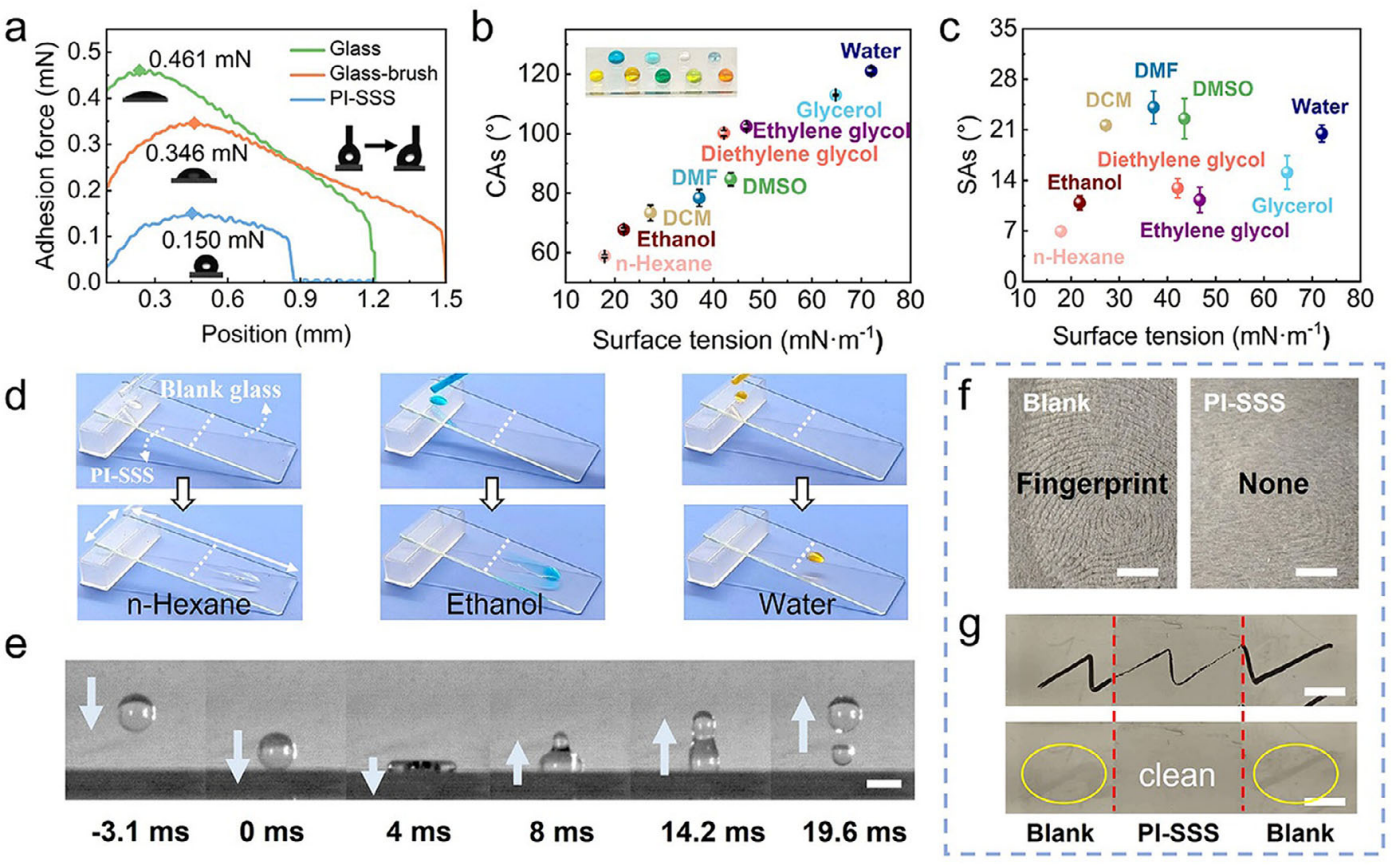
Lubricating and bouncing properties of the PI‐SSS. a) Water contact angle and adhesion of water droplets (4 µL) on different substrates (blank, brushes, and PI‐SSS). b) CAs of the liquids (4 µL) with different surface tensions on PI‐SSS. c) SAs of different surface tension liquids (10 µL) on PI‐SSS. d) Sliding behavior of n‐hexane, ethanol, and water on the PI‐SSS and blank glass (with a droplet of 20 µL and tilted angle of 30°) (glass size: 75 mm × 25 mm). e) Time‐resolved images of the bouncing of a 5 µL water drop on the PI‐SSS (bar scale: 2 µm). The PI‐SSS coated 316L SS demonstrated anti‐adhesion properties to fingerprint f) and ink g) (bar scale: 3 µm).

The liquid repellency and low adhesion properties of the PI‐SSS were evaluated through water contact angle (CAs) and the adhesion test. Due to perfluoroalkyl groups providing ultra‐low surface energy via hydrophobic interaction,^[^ ^25^ ^]^ PFB copolymer coated on the brush layer resulted in an increase in the CAs from 34.5° to 121.1°, obtaining a hydrophobicity surface (Figure S9, Supporting Information). Correspondingly, the adhesion force of water droplets (4 µL) decreased from 0.461 mN on the bare surface to 0.150 mN on the PI‐SSS surface (**Figure** **3**a). Further investigations with CAs for 4 µL droplets and sliding angle (SAs) for 10 µL droplets measurements demonstrate the wettability and sliding behavior of different liquids on the PI‐SSS (n‐Hexane (17.9 mN·m^−1^), Ethanol (22.3 mN·m^−1^), DCM (27.2 mN·m^−1^), DMF (37.1 mN·m^−1^), Diethylene glycol (42.1 mN·m^−1^), DMSO (43.5 mN·m^−1^), Ethylene glycol (46.7 mN·m^−1^), Glycerol (61.9 mN·m^−1^), and water (72.8 mN·m^−1^)). The CAs increase as the surface tension increases, and all liquids remain spherical and stability without deforming. Even n‐Hexane, with the lowest surface tension, did not wet or slump on the PI‐SSS (Figure 3b inset). By tilting the PI‐SSS coated surface at a small angle, all fluids exhibited straight‐line sliding behavior with no drag (Figure 3c; Figure S10, Supporting Information). In contrast, the liquids (n‐Hexane, ethanol, and water) converted to a wetted state on the blank glass, leaving a residual liquid on the surface (Figure 3d). These results show that the PI‐SSS coating imparts excellent slippery properties to the material surface.

Specifically, the slippery property of the PI‐SSS facilitates the liquid's ability to rebound, thereby preventing adhesion and the formation of residual contaminants.^[^ ^26^ ^]^ As captured by the high‐speed camera, the water droplets contacted the surface and subsequently spread out evenly at 4 ms, transforming a sphere into a pancake‐like shape (Figure 3e). The retraction force then caused them to regain their spherical shape, which led to the achievement of the highest point at 19.6 ms. Meanwhile, toluene and ethylene glycol also showed popping ability (Movie S1, Supporting Information).

The exceptional slippery properties of the PI‐SSS endow it with unique self‐cleaning capabilities. We chose various solids with different chemical properties (soil, SiO_2,_ and methyl orange powders) and common household liquids (seawater, tea, whiskey, orange juice, and vinegar) as contaminants. Compared with blank and brush coated substrates (glass and 316 SS), the PI‐SSS rapidly slid off exposed clean surfaces (Figure S11, Supporting Information). Specifically, crude oil,^[^ ^27^ ^]^ a viscous mixture, left minimal residue on the PI‐SSS surface, which could be easily removed with a mild airflow. The PI‐SSS also showed multifunction antibiofouling properties, including anti‐fingerprint and anti‐ink (Figure 3f, g). The oceans are abundant in energy and mineral resources, yet the presence of numerous natural pollutants poses significant challenges to their exploitation, such as mussels and algae.^[^ ^28^ ^]^ The anti‐bioadhesion performance of PI‐SSS was evaluated using mussel growth solution, Chlorella cultures, and protein. The results indicated that mussels and protein had difficulty adhering to the PI‐SSS surface (Figures S12 and S13, Supporting Information), and Chlorella coverage remained minimal even after a 30‐day immersion period (Figure S14, Supporting Information).

### 2.3 Thermally Induced Self‐Healing and Durability

**Figure 4:**
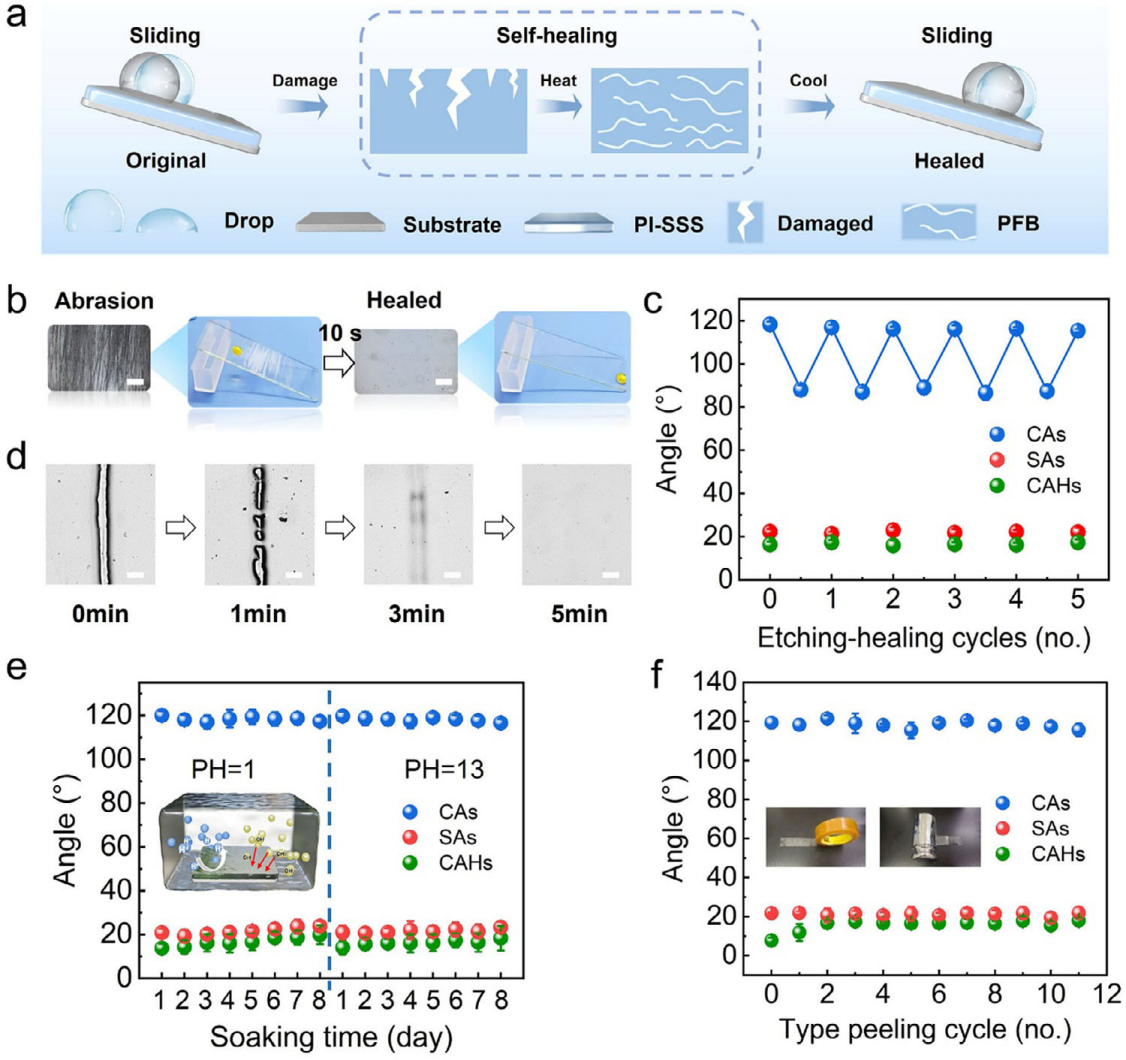
Thermally induced self‐healing properties and stability of PI‐SSS. a) Schematic of restored droplet sliding behavior on damaged PI‐SSS surfaces after thermally induced self‐healing. b) Pinning and sliding behavior of water droplets on physically damaged and self‐healed PI‐SSS surfaces, along with microscopic images of the PI‐SSS coated glass substrate (bar scale: 200 µm). d) Microscopic images of PI‐SSS on a glass substrate showing thermally assisted healing of physical damage (bar scale: 200 µm). CAs, SAs, and CAHs of the PI‐SSS after multiple cycles of oxygen plasma etching (5 min) and thermally induced self‐healing c), as well as acid‐base corrosion time (pH = 1 and pH = 13) e), tape stripping failure f).

The self‐healing capability of the coating is a crucial factor for its practical application. Once degraded, the inherent liquid repellency of many hydrophobic materials is lost, since they form a rough surface that pins liquid droplets. PFB copolymer, a crystalline thermoplastic material, can undergo a solid‐liquid transition at different temperatures.^[^ ^19^ ^]^ In its liquid state, PFB polymer chains interdiffuse to allow the material to refill damaged areas and restore the slippery properties of the PI‐SSS coating (**Figure** **4**a).^[^ ^29^ ^]^ Optical microscopy and photographs revealed that the coating heals rapidly when heated to 100 °C for 10 s after being rubbed with 2000 grit sandpaper (Figure 4b). Subsequently, water droplets which pinned to damaged surfaces slough off following thermally induced self‐healing. Even after 50 abrasion‐healed cycles, the CAs, SAs, and contact angle hysteresis (CHAs) of the PI‐SSS coating changed very slightly (Figure S15, Supporting Information). Furthermore, thermally induced self‐healing can also restore the slippery surface property lost after oxygen plasma etching, which raises the surface energy (Figure 4c; Figure S16, Supporting Information).^[^ ^30^ ^]^ To further investigate the mechanism of thermal‐assisted self‐healing, a scratch with a depth of 3.15 ± 0.67 µm was made on the PI‐SSS surface with a blade (Figure S22, Supporting Information). Upon heating at 100 °C, the molten PFB flowed rapidly into the damaged area forming a smooth liquid layer via capillary forces, which underwent solidification forming a slippery coating (Ra = 0.904 nm, Rq = 1.13 nm) as it cooled to room temperature (Figure S17, Supporting Information). In addition, the complete healing times varied depending on the substrate, with healing completed in approximately 10 minutes on PE, 8 minutes on PET, and 5 minutes on glass, 316 SS, and Al_2_O_3_ substrates (Figure 4d; Figure S23, Supporting Information).

To assess the operational stability of PI‐SSS under realistic conditions, we performed accelerated degradation tests employing both chemical and physical stressors to simulate environmental exposure. The pure Al_2_O_3_ surface exhibited significant corrosion under extreme pH conditions (pH = 1 and pH = 13), while the PI‐SSS coated Al_2_O_3_ surface remained intact along with water droplets rapidly sliding off within 0.2 s (Figure S18, Supporting Information). After acid and alkali immersion cycles, there was no significant change in CAs (≈120°) and SAs (≈21°), indicating excellent acid and alkali resistance of the PI‐SSS (Figure 4e). In 50‐cycle adhesion/peeling tests that mimic basic adhesion in nature, the CAs only marginally decreased; meanwhile, PFB consistently maintained an effective binding with the substrate (Figure 4f). Moreover, the PI‐SSS maintained excellent liquid repellency after cyclic high‐low temperature (‐15 °C to 100 °C, 50 cycles) and soaking in seawater for 50 days (Figures S19a and S20, Supporting Information). Meanwhile, the PI‐SSS significantly delayed the freezing time (397s) (Figure S19b, Supporting Information), reduced ice adhesion on glass (≈0.206 MPa) (Figure S19c, Supporting Information) and exhibited minimal lubricating copolymer loss (0.018 ± 0.055 mg·cm^−2^) (Figure S19d, Supporting Information), demonstrating the PI‐SSS's excellent anti‐icing and high interfacial stability. Notably, UV radiation can cause some damage to the coating, but this will be restored after thermal repair (Figure S20, Supporting Information). All in all, the PI‐SSS is anticipated to be employed in natural environments and function over an extended period.

### 2.4 Coating on Photovoltaic Panels

**Figure 5:**
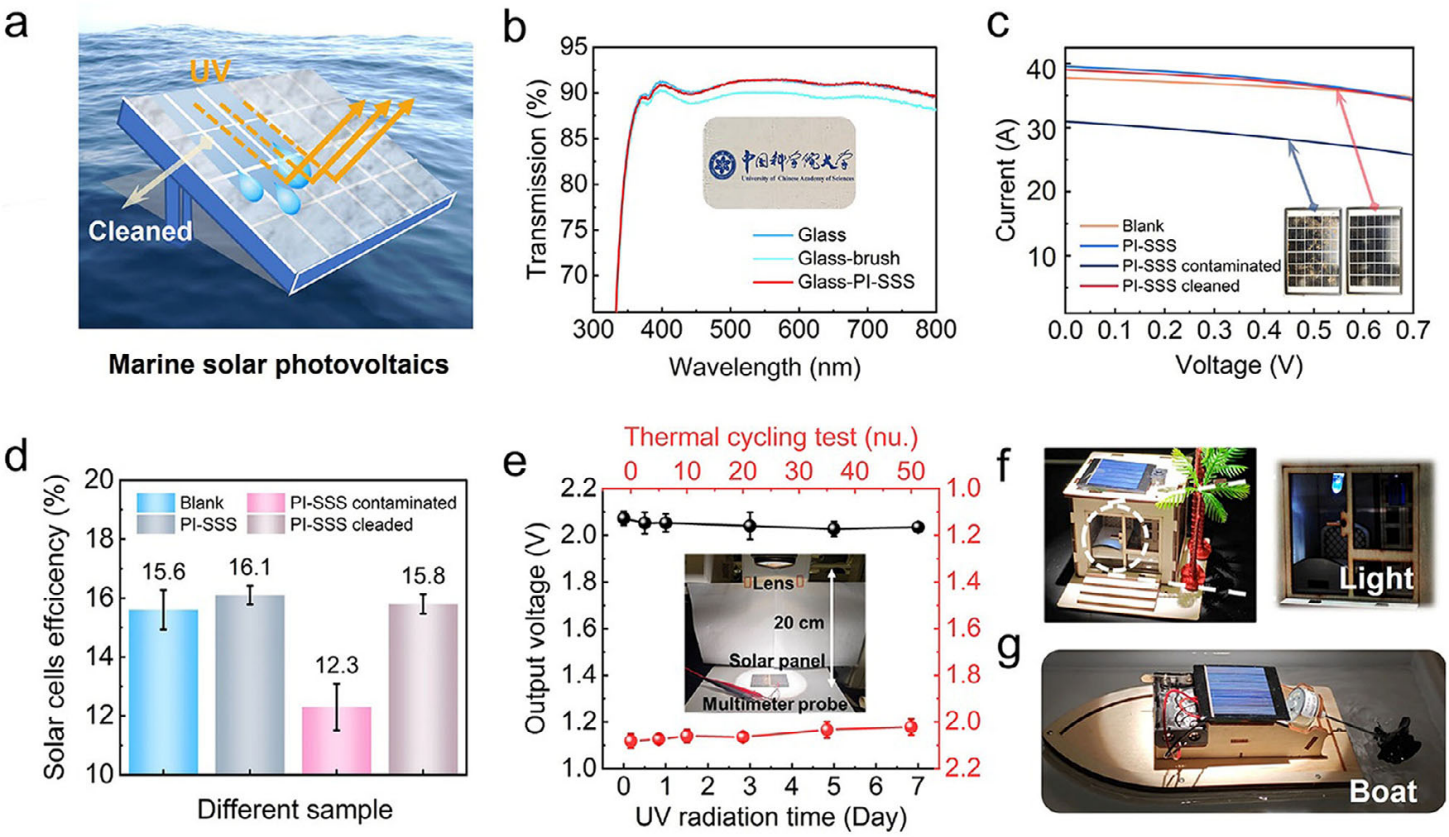
Performance of solar cells after PI‐SSS coatings. a) Schematic diagram of the PI‐SSS coated solar panel to protect from destruction. b) Optical transmission measurement for PI‐SSS coated glass in the visible light range (300–800 nm). Current intensity‐voltage (I‐V) curves c) and power conversion efficiencies d) of blank and PI‐SSS coated solar cells under different conditions (pristine, contaminated, and cleaned). e) Stability evaluation of output voltage for PI‐SSS coated panels under accelerated aging tests: continuous UV irradiation (365 nm, 7 days) and thermal cycling (‐15 °C to 100 °C, 50 cycles). Demonstration of practical applications using PI‐SSS coated solar panels for residential lighting f) and marine power systems g).

Coastal areas have been identified as optimal locations for the deployment of solar cells (**Figure** **5**a).^[^ ^31^ ^]^ The optical transmittance of the coating is one of the key factors affecting the conversion efficiency of solar panels.^[^ ^32^ ^]^ The PVBImBF₄ brush coated glass shows reduced transmittance (≈90.0%) due to increased surface roughness (Ra ≈ 1.37 nm, Rq ≈ 1.62 nm), which promotes interfacial light scattering (Figure 5b; Figures S17, Supporting Information). In contrast, the PI‐SSS coated glass exhibits excellent transmittance (≈91.3%) in the visible range (300–800 nm), indicating its light‐transmission‐enhancing property. The photovoltaic performance of solar cells is significantly influenced by environmental contaminants (e.g., dust), resulting in diminished light absorption and energy conversion efficiency. Due to the self‐cleaning properties of PI‐SSS, the coating can provide effective protection for solar panels against dust pollution (Figure 5c inset). Under illumination from a xenon arc lamp (1500 W/m^2^) (simulated AM1.5 spectrum), the reference solar panel (standard specifications: 2.5 V, 130 mA) demonstrated an open‐circuit voltage (*V_oc_*) of 2.09 V and a short‐circuit current (*I_sc_*) of 39.51 mA (Figure 5c). The PI‐SSS coated panel showed comparable performance (*I_sc_* = 39.07 mA), confirming minimal interference with photovoltaic function. When contaminated with dust, this panel's *I_sc_* decreased significantly to 30.95 mA (≈21.7% reduction), but subsequent self‐cleaning functionality restored its output to baseline levels, demonstrating complete recovery of photovoltaic performance. The power conversion efficiency (PCE) of the solar cells was restored from 12.3% to 15.8% following the self‐cleaning process (Figure 5d). To assess the protective effect of PI‐SSS coating on solar panels, we conducted accelerated aging tests consisting of: (i) 7 days continuous UV irradiation (365 nm) and (ii) 50 thermal cycles between ‐15 °C and 100 °C (Figure 5e). Remarkably, the coated solar panels exhibited only a slight decrease in output voltage, which remained above 2.0 V throughout. Meanwhile, the damaged PI‐SSS coated solar panels continue to exhibit illumination capability (Figure 5f) and sustained operation of a connected DC motor (Figure 5g). These results demonstrate that PI‐SSS holds significant practical value for extreme marine photovoltaic protection.

## 3 Conclusion 

Herein, we show the design and fabrication of a plant‐inspired solid slippery surface (PI‐SSS) by mimicking plant cuticle structure. This strategy relies on a brush matrix to capture lubricant copolymer via ion‐dipole interactions (≈0.96 MPa, adhesion strength), forming the slippery layer on various substrates. The resulting PI‐SSS exhibits excellent transparency (≈91.3%) and repellency toward diverse drinks (e.g., tea, whisky, orange juice, vinegar, etc.), living pollutants (e.g., seawater, crude oil, fingerprint, ink, etc.), and organisms (e.g., algae, mussels). Due to the crystalline thermoplastic nature of the lubricating copolymer, the slippery surface is equipped with thermally induced self‐healing capability to recover sliding properties after continuous abrasion and oxygen plasma etching. Meanwhile, the surface displays chemical stability, anti‐icing, and antifouling ability against contaminants such as fingerprint, ink, crude oil, and mussels. These functionalities enable the PI‐SSS coated solar panels to withstand extreme environments including continuous UV irradiation for a week and 50 thermal cycles between ‐15 °C and 100 °C, while maintaining a stable voltage output of 2.0 V, demonstrating an efficient strategy for offshore photovoltaic protection.

## Conflict of Interest

The authors declare no conflict of interest.

## Data Availbility Statement

The data that support the findings of this study are available from the corresponding author upon reasonable request.

## Supporting information



Supporting Information

Supplementary Movie S1
