# Supplementary material for: Bioinspired Brush Reinforced Solid Slippery Coatings for Marine Photovoltaic Protection
Source: Adv Sci (Weinh). 2025 Jun 10;12(40):e05526. doi: 10.1002/advs.202505526 (PMC12561401; doi:10.1002/advs.202505526)
Supplement: Supplementary file 1 — Supporting Information [file ADVS-12-e05526-s002.docx]

**Supplementary Information**

**Bioinspired Brush Reinforced Solid Slippery Coatings for Marine Photovoltaic Protection**

Ling Yin, Runxiang Tan, Junyi Han, Jianing Wang, Jianjun Cheng, Daheng Wu*, Tao Zhang*, and Liping Wang*

Ling Yin, Junyi Han, Jianing Wang, Jianjun Cheng, Daheng Wu, Tao Zhang, Liping Wang

State Key Laboratory of Advanced Marine Materials, Ningbo Institute of Materials Technology and Engineering, Chinese Academy of Sciences, Ningbo 315201, China

E-mail: [wangliping@nimte.ac.cn](mailto:wangliping@nimte.ac.cn); [tzhang@nimte.ac.cn](mailto:tzhang@nimte.ac.cn); [wudaheng@nimte.ac.cn](mailto:wudaheng@nimte.ac.cn)

Ling Yin

University of Chinese Academy of Sciences, Beijing 100049, China

Runxiang Tan

College of Material Science and Engineering, Sichuan University, Chengdu 610064, China

1. **Experimental Section**
   1. **Materials**

Cu plate (0.05 mm, purity >99.99%) was obtained from Beijing ZhongNuo Advanced Materials Technology., Ltd. Silicon wafers with an oxide layer (~300 nm) were purchased from Suzhou RuiCai Semiconductor Co., Ltd. Gold chips were purchased from Biolin Scientific with SX 301. Solar panels obtained from Shenzhen Beike Trading (54 cm × 54 cm, 2.5 V × 130 mA). 1,1,4,7,7-pentamethyldiethylentriamin (PMDETA) were purchased from Sigma-Aldrich. 2-(Perfluorooctyl)ethyl methacrylate (PFEMA, purity ≥ 99%), 2,2'-Azobis(2-methylpropionitrile) (AIBN), methanol (MeOH, purity ≥ 99.8 %), Tris[2-(dimethylamino) ethyl] amine (Me_6_TREN), Toluene dimethyl sulfoxide (DMSO, purity ≥ 99.8 %), N,N-Dimethylformamide (DMF, purity ≥ 99.9%), diethylene glycol, ethylene glycol glycerin, and phosphate buffer solution (PBS) were obtained from Shanghai Aladdin Biochemical Technology Co., Ltd. Butyl Methacrylate (BMA) and dichloromethane (DCM) were obtained from Sinopharm Chemical Reagent. 1-butyl-3-vinyliMidazoliuM tetrafluoroborate (VBImBF_4_, purity ≥ 99%) were purchased from Anhui Senrise Technologies Co., Ltd. 3-Trimethoxysilylprooyl2·Bromo-2-Methylpropoinate as an initiator were purchased from Shanghai McLean Biochemical Technology Co., Ltd. Tluorescently labeled bovine serum albumin (BSA) and red blood cell (5%) was purchased from Beijing Dabono Technology Co., Ltd. Mussel was purchase from aquatic product stores. Deionized water was used throughout the experiments and tests. The model of the DC motor is JQ24-35H440.

- 1. **Self-assembled monolayer (SAM) of initiator on substrate**

Silicon wafer with a nanoscale oxide layer (or glass, stainless steel 316L (316L SS), aluminum oxide (Al_2_O_3_), polyethylene (PE), and polyethylene terephthalate (PET)) was washed alternately with methanol and deionized water for 3 min and then ionized by oxygen plasma (PEC-6, 600 W, Sykejing company) for 600 s. Then, the substrates were functionalized with 5 μL 2-bromo-2-methyl-6-trimethoxysilylhexanoate1^[1]^ in a closed container by vapor deposition for 3 h at 60 ℃. After that, the functionalized substrates were rinsed with deionized water and ethanol then dried with a dry nitrogen flow.

- 1. **Synthesis of PVBImBF_4_ polymer brushes**

Mixing concentrations 500 μL VBImBF_4_, 1 mL water, 0.5 mL methanol, and 37 μL PMDETA to obtain the polymerization solution. The Cu sheet was washed with a mixed solution of 3M HCl and methanol (v: v = 1: 1) under ultrasonication for 3 min and dried with a nitrogen stream. Next, the Cu sheet and the initiator functionalized substrate were placed in parallel with a distance of 0.20 mm. The assembly was submerged in the polymerization solution and taken out after polymerizing for a period of time at room temperature via surface-initiated copper-mediated controlled radical polymerization (SI-Cu^0^CRP)^[2]^ (1 h unless otherwise specified). After that, the substrate was washed with water and ethanol, and finally dried under nitrogen flow to obtain polymer brushes.

- 1. **Synthesis of Poly(PFEMA)-co-poly(BMA) (PFB)**

Weigh 4 g of PFEMA, 0.53 g of BMA, 45.3 mg of AIBNs, and 20 mL of DMF, and mix them uniformly in a 100 mL round-bottom flask. Deoxygenate the mixture by bubbling with N_2_ for 30 minutes while stirring at 400 rpm. Subsequently, the mixed solution was heated at 65 °C for 24 hours for polymerization, while continuously stirring. After the reaction, a white solid precipitate was obtained^[3]^. This was thoroughly washed with ether and then placed in a vacuum drying oven, where it was dried at room temperature for 6 hours, yielding 3.48 g of Poly(PFEMA)-co-poly(BMA) (PFB), with a yield of approximately 77%.

- 1. **Synthesis of PI-SSS**

Based on the substrate area, a measured amount of PFB powder (0.107 ± 0.012 mg·cm⁻²) was applied to the surface. The substrate was subsequently heated at 100 °C on a hot plate, and the powder was spread uniformly using applicators (a solvent-free coating method). The coated sample was then cooled and fixed for further use.

- 1. **Characterizations**
     1. **General characterization**

Scanning electron microscopy (SEM) images were obtained using a hot field high-resolution scanning electron microscope (GeminiSEM 300) at an acceleration voltage of 5 kV. The elemental mapping images were obtained from a BRUKER XFlash 6I100 at an acceleration voltage of 20 keV and a current of 10 μA. Scanning probe microscopy (SPM) images were recorded from a 143 Dimension ICON SPM (Bruker, America) in the tapping mode. Micro-Fourier transform infrared spectroscopy (FRIT) was performed using a Cary660 + 620 (Agilent, America) in the range of 400-4000 cm−1. X-ray photoelectron spectroscopy (XPS) was obtained from Axis DLD (Kratos, England). Static water contact angle, slide water angle and contact angle hysteresis measurements were carried out with DCAT21 (Ningbo Jin Mao Import & Export Co., Ltd). The Transmittance was measured by the UV-Vis spectrophotometer (Lambda 950, Perkin-Elmer, USA) in the range of 200-800 cm-1. The fluorescence images were obtained by a fluorescence microscope (Zeiss Axio Observer Z1m, Zeiss, Oberkochen, Germany) and the coverage areas were calculated by ImageJ. The liquid nuclear magnetic resonance (NMR) spectroscopy was measured on the Bruker AVANCE 400/600 WB spectrometer (400 MHz for 1H).

- - 1. **Coating-substrate adhesion tests**

Shear force tests were performed using a 1 KN universal material testing machine (Zwick/Roell Z1.0 model, Shanghai). Firstly, PVBImBF_4_ was grafted on half the area of the slide, and then heated PFB was evenly coated on the surface to form a liquid layer. Next, another equally treated slide was pressed onto the liquid layer and cooled to room temperature to allow the two sides to bond through the solidification of the liquid layer. Afterwards, a gradually increasing tensile force was applied to these bonded slides until separation. At the same time, polymer samples that were not treated with the liquid layer were prepared as controls.

- - 1. **Protein adhesion assays**

The protein adsorption assay was adapted from a previous report by Yang et al^[4]^. First, the prepared testing samples (10 mm × 10 mm) were sterilized under ultraviolet light for 1 h and equilibrated for 2 h in phosphate buffer solution (PBS, pH = 7.4). Subsequently, the samples were immersed in prepared BSA solutions (1 mg/mL) to incubate at 37 °C and 200 rpm for 4 h. Each sample was gently washed three times with PBS and dried by nitrogen stream. Finally, the confocal laser scanning microscope was used to image the amount of attached protein.

- - 1. **Algae settlement and adhesion assays**

Phaeodactylum tricornutum and Chlorella were selected for algal adhesion assays. The prepared testing samples were immersed in Phaeodactylum tricornutum and Chlorella suspensions (the cell concentrations were approximately 8×10^6^ cells mL^-1^), respectively, and cultured at 25 °C for 1 day and 30 days (12 h light and 12 h dark cycles). Then, the samples were rinsed with deionized water after being taken out, dried with nitrogen stream. An optical microscope and confocal laser scanning microscope were used to image the amounts of attached algae, and ImageJ was used to determine the algae intensity from the obtained micrographs. The average value and standard deviation of the algal coverage were calculated for each collected image.

- - 1. **Molecular dynamics simulation**

To further explore the interaction between PVBImBF_4_ polymer brush and Poly(PFEMA)-co-poly(BMA) (PFB), we calculated the adsorption energy (*E_adsorption_*) of PVBImBF_4_ brushes or substrate (SiO_2_) with PFB copolymer: solid-solid interface.

$E_{absorption}=E_{ab}-E_{a}-E_{b}$ (1)

where *E_ab_* is the total energy of the system including the brush and copolymer, *E_a_* is the energy of the system with one brush or substrate, and *E_b_* is the energy of the system with PFB copolymer, respectively. The negative binding energy usually indicates an energetically favorable adsorption between the adsorbate and the target surface.

First, the Amorphous Cell modeling was used to build the model of polymer brush and Poly(PFEMA)-co-poly(BMA) (PFB), and the density of PVBImBF_4_ was 0.1 g·cm^-3^. Then the MD simulation was performed using GROMACS 2020.6 package.^[5]^ The parameters for the cross atoms were obtained using the Lorentz–Berthelot mixing rules. Simulation boxes were constructed with polymers in a box. The systems were simulated under constant pressure (1 bar) and constant temperature (298.15 K) conditions using the Berendsen (τp = 0.5 ps) and V-rescale thermostat (τt = 0.2 ps). H-bonds were constrained by using the LINCS algorithm. Both van der Waals and coulomb radius cutoff were set as 1.2 nm and the long-range electrostatic interaction was calculated using the particle mesh Ewald (PME) method. An integration time step of 2 fs was used. After relaxation of the initial conformation of the system in GROMACS energy was minimized using the steepest descent algorithm until convergence and then equilibrated in the canonical ensemble (NVT) for 100000 steps and then in the isothermal–isobaric ensemble (NPT) for 100000 steps. The final 10 ns trajectories were used for post-analysis and visualized by visual molecular dynamics (VMD) 1.9.3.^[6]^

- - 1. **DFT calculation**

All electronic structure calculations were carried out using the Gaussian 16 software package. The molecular geometries of all investigated systems were fully optimized at the B3LYP-D3(BJ)/def2-TZVP level of theory. Harmonic vibrational frequency analyses were performed at the same level to confirm that the optimized structures correspond to true minima (no imaginary frequencies).

To gain insight into the nature of weak intermolecular interactions, the wavefunction files generated from Gaussian were subsequently analyzed using the Multiwfn program.^[7]^ Non-covalent interaction (NCI) analysis was conducted by calculating the reduced density gradient (RDG) function. The resulting RDG isosurfaces were plotted and colored based on the sign of the second eigenvalue (λ₂) of the electron density Hessian multiplied by the electron density [sign(λ₂)ρ], which allows for visual identification and qualitative assessment of different interaction regions.^[8]^ In this representation, attractive interactions such as hydrogen bonding and van der Waals forces appear as blue or green regions, while steric repulsion is indicated by red regions.

- - 1. **Ice adhesion strength test**

The ice adhesion strength was measured using an INSTEC-ZTS (500 N) device. Samples were placed horizontally and cooled to −20 °C. Deionized water (0.5 mL) was dropped onto the sample surface and frozen for 2 hours to form an ice column. The frozen samples were then subjected to a displacement rate of 0.2 mm/s until the ice detached. The maximum force recorded during detachment was used to calculate the ice adhesion strength (MPa).

- - 1. **Water adhesion test**

The adhesion force measurements in Figure 3a were performed using a DCAT21 contact angle and tensiometry system (Ningbo Jin Mao Import & Export Co., Ltd). A 4 μL water droplet was dispensed from a needle onto the horizontally mounted sample surface. The needle was then retracted vertically at a constant rate of 0.2 mm/s, and the force required to detach the droplet was recorded. The peak force during this process was defined as the adhesion force.

- - 1. **Wettability test**

Static contact angles (CAs), sliding angles (SAs), and contact angle hysteresis (CAHs) were measured using a DCAT21 analyzer (Ningbo Jin Mao Import & Export Co., Ltd) under ambient conditions (~20°C).

CAs: Droplets (5, 10, and 20 μL) were deposited on the horizontal sample surface. After stabilization, the left and right contact angles (θ₁ and θ₂) were measured, and the static contact angle was calculated as:

$CAs=\frac{\theta_{1}+\theta_{2}}{2}$ Equation S1

SAs: After droplet deposition, the sample stage was tilted, and the angle at which the droplet started to move was recorded.

CAHs: The advancing (θₐ) and receding (θᵣ) contact angles were recorded during droplet movement, and CAHs was calculated as:

$CAHs=\theta_{a}-\theta_{r}$ Equation S2

- - 1. **Output voltage test of solar panels**

The stability of the solar panels was evaluated using a two-electrode system with a KEITHLEY digital source meter. Briefly, the positive and negative terminals of the solar panel were connected to the corresponding electrodes of the source meter. Under illumination from a xenon arc lamp (1500 W/m²) (simulating the AM1.5 solar spectrum), the output voltage was continuously recorded over 5 minutes to assess device stability.

- 1. **Statistical Analysis**

All values are expressed as the mean ± the standard error of the mean (s.e.m). Data was analyzed using Origin 2024 software.

1. **Supplementary Figures**


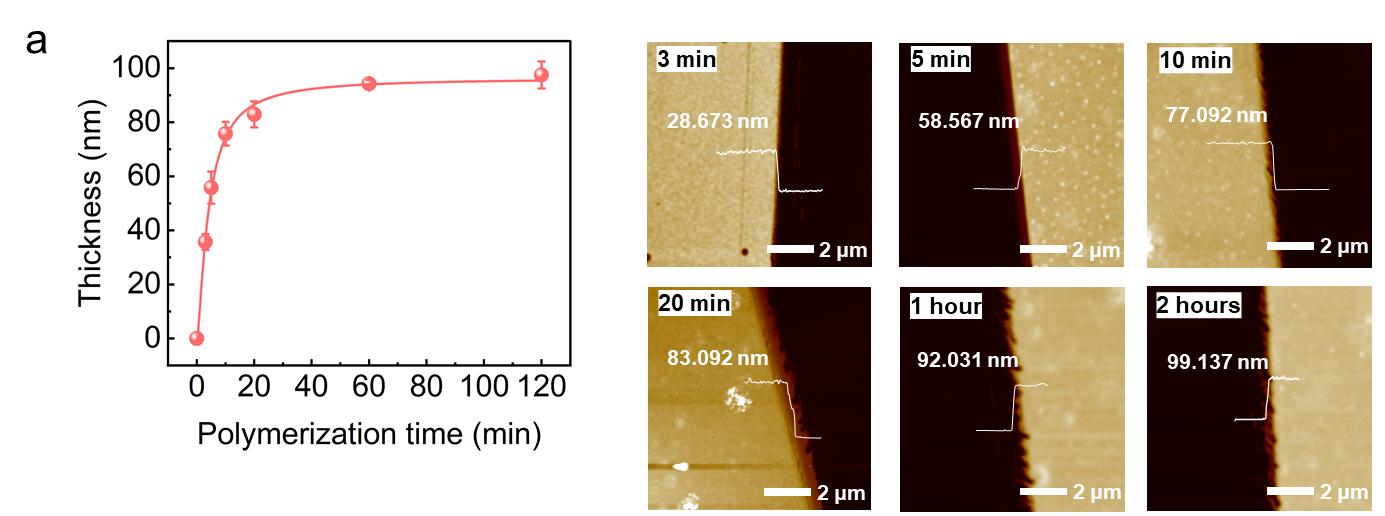


1. (a) Variation of the growth thickness of PVBImBF_4_ brushes with polymerization time (3 min, 5 min, 10 min, 20 min, 1h, and 2 h). (b) Scanning probe microscope topographic scan and high profile taken at the PVBImBF_4_ brushes layer.

The length of the polymer brushes is a key factor in determining the content of imidazole groups, which in turn affects the bond strength of the coating. Therefore, it is crucial to thoroughly investigate the growth kinetics of polymer brushes. As shown in Figure S1, polymer brushes incubated with 500 μL of VBImBF_4_ solution exhibited the maximum thickness as found by SPM testing. In addition, the thickness of the polymer brushes increased over time, with a high growth rate during the first 20 min, while the thickness stabilized at 99.137 ± 2.17 nm after 1 h. Based on these results, it can be determined that 500 μL of VBImBF_4_ solution and 1 h of incubation time are the optimal conditions for the growth of PVBImBF_4_ brushes. Unless otherwise stated, subsequent experiments will be conducted using these optimal conditions for experimental efficiency.


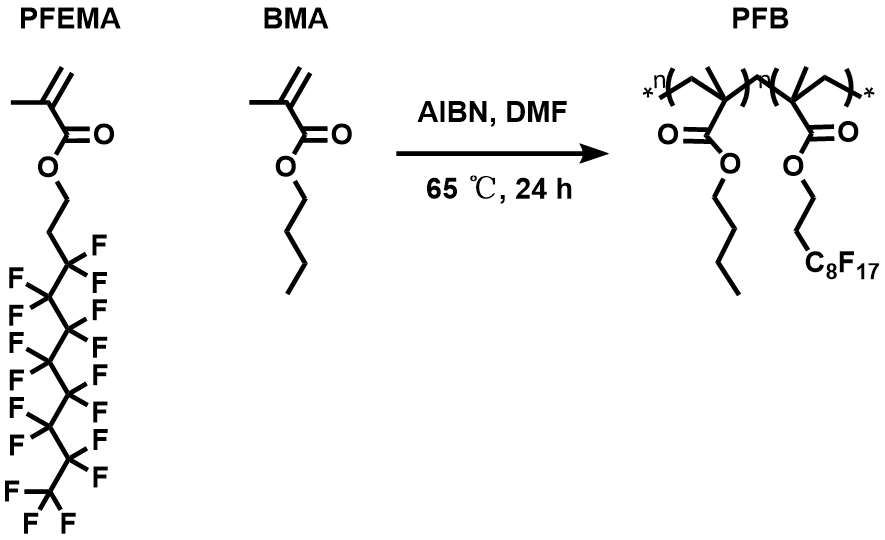


1. Schematic of the synthetic route of the poly(PFEMA)-co-poly(BMA) copolymer (PFB).


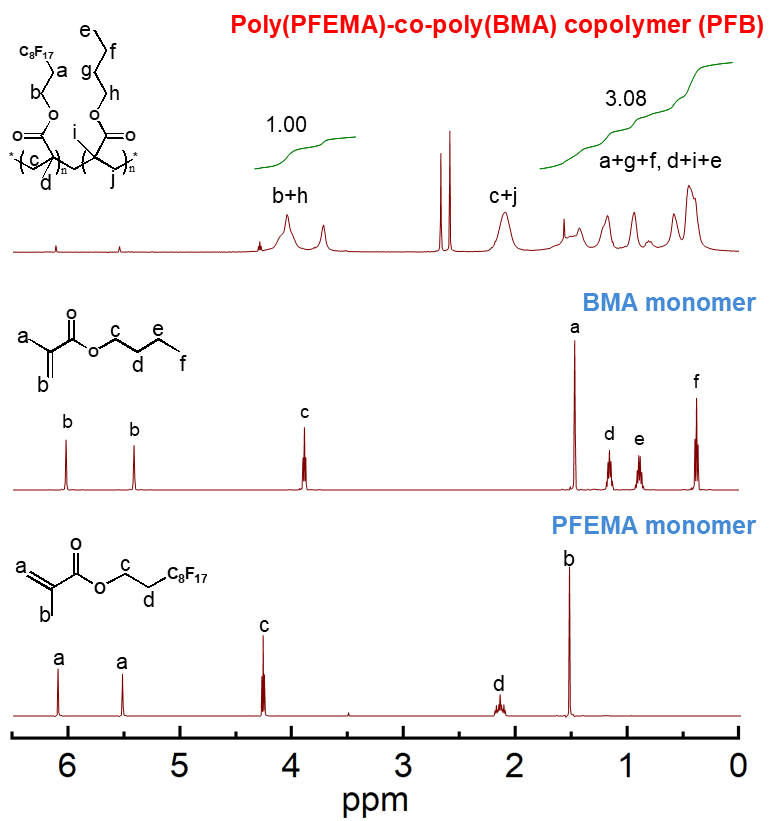


1. ^1^H NMR (400 MHz) spectra of the poly(PFEMA)-co-poly(BMA) copolymer, and the BMA and PFEMA monomers.

Solid (Poly(PFEMA)-co-poly(BMA)) (PFB) copolymers with low surface energy were obtained by free radical polymerization of PFEMA and BMA. At appropriate melting temperatures, PFB is a crystalline thermoplastic material that can be easily modified on large-area substrate surfaces using a solvent-free scratch-coating technique to form a transparent, solid, and smooth coating. According to the 1H pattern of PFB, the A peak of PFB is contributed by two H atoms at the h position of the BMA unit and five hydrogen atoms at the a and d positions of the PFEMA unit, and thus the molar ratio between PFEMA and BMA in the PFB copolymers can be calculated by Equation (2) and (3):

2n_BMA_+2n_PFEMA_=1 (2)

10n_BMA_+5n_PFEMA_=3.04 (3)

The molar ratio between PFEMA and BMA in the PFB copolymer was calculated to be 3.63 : 1.00.


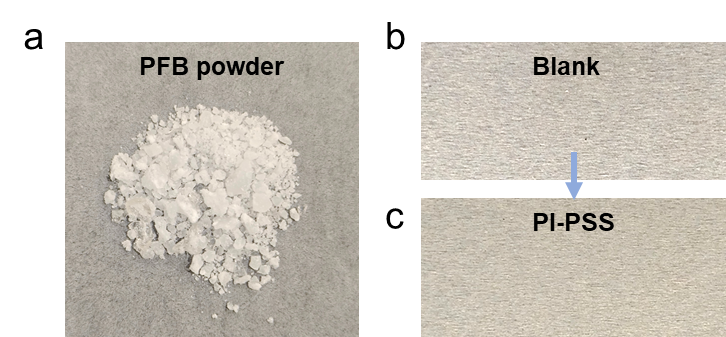


1. Photographs of the optical morphology of PFB copolymer (in powder form) and PI-SSS are shown. The PFB modification of the 316 SS surface had a negligible effect on the morphology of the substrate.


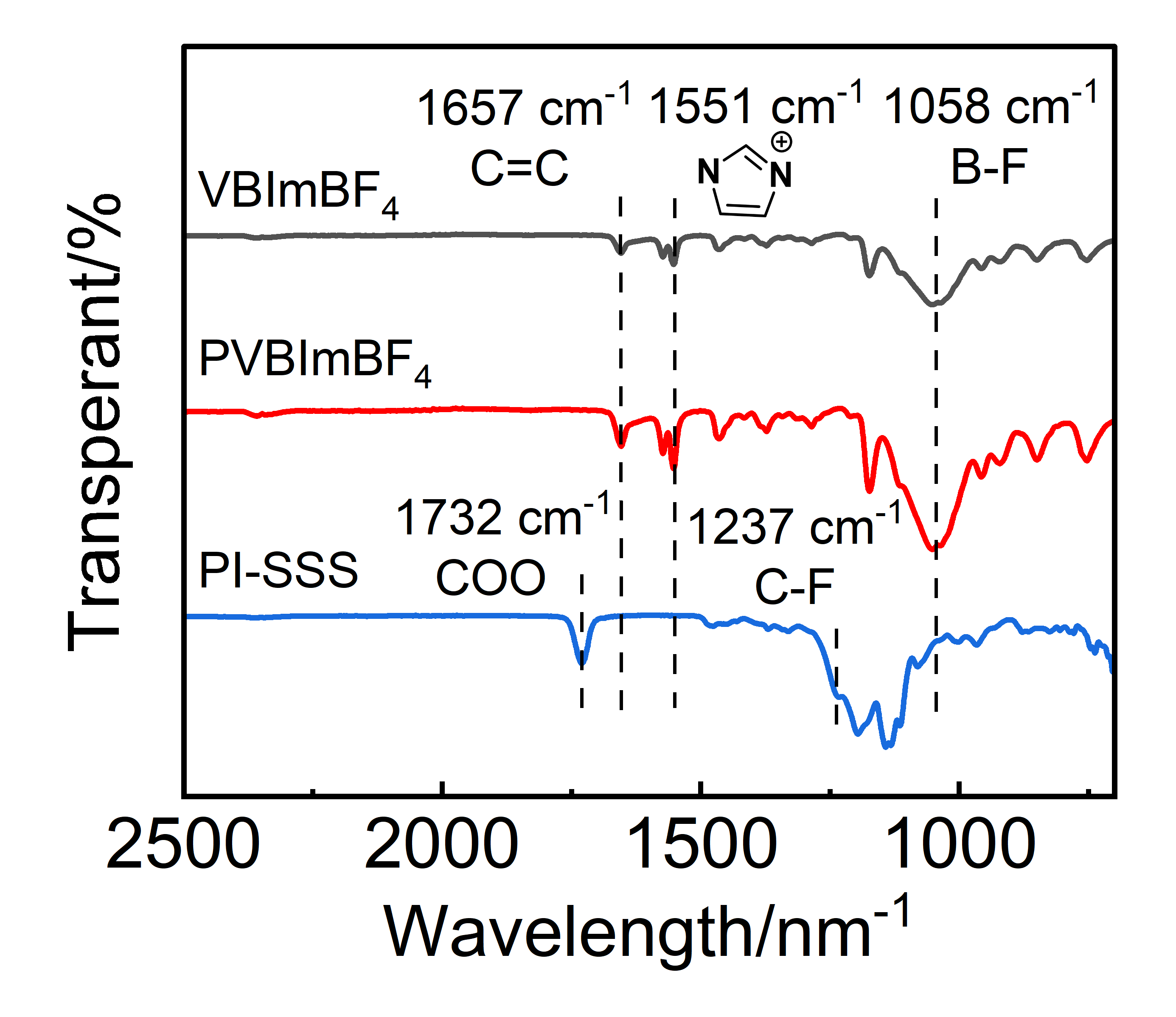


1. The infrared spectra of different materials, including VBImBF_4_ monomer, PVBImBF_4_ brushes, and PI-SSS coating.


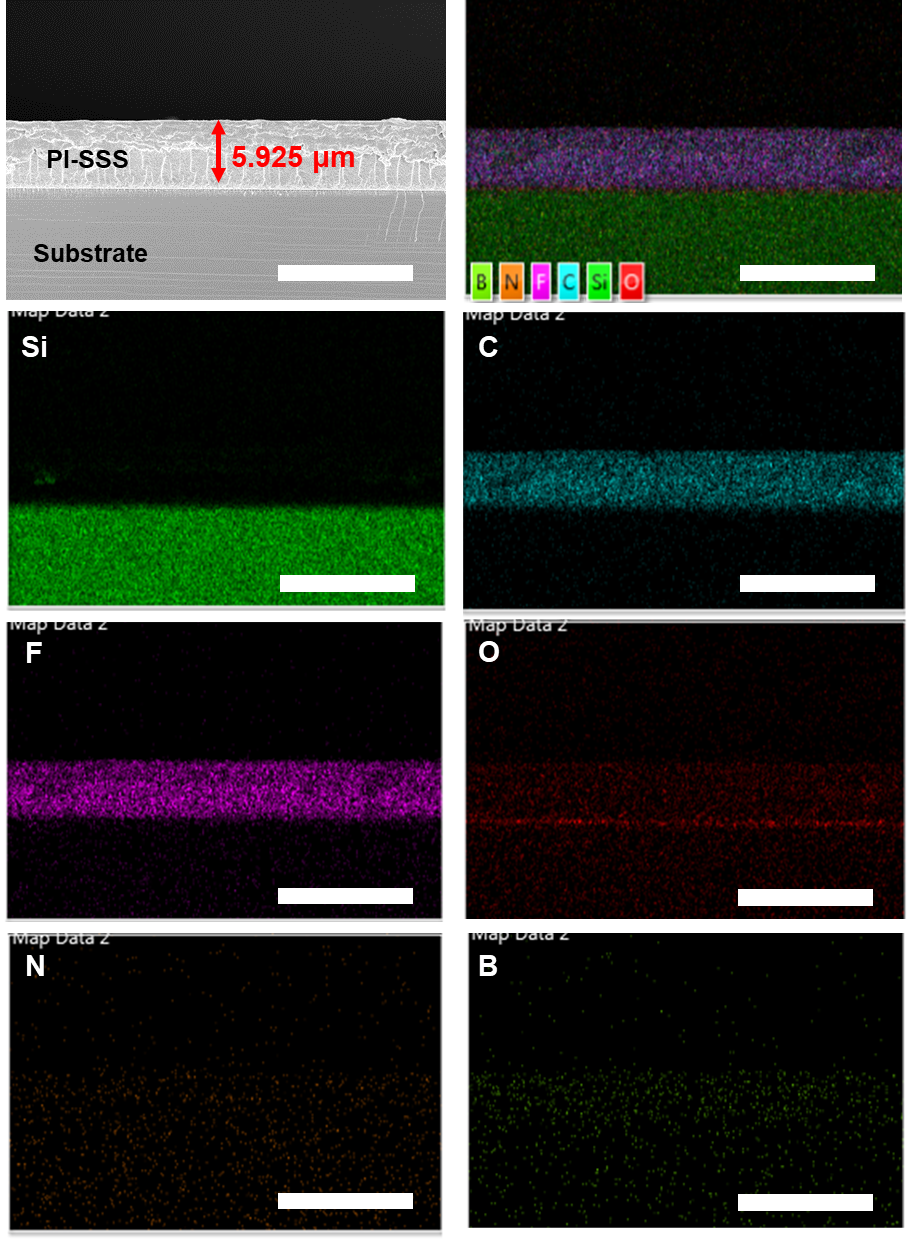


1. SEM images and EDS spectra of PI-SSS (the elements include Si, C, F, O, N, and B). Scale bar: 10 μm. The height of PI-SSS is about 5.925 μm.


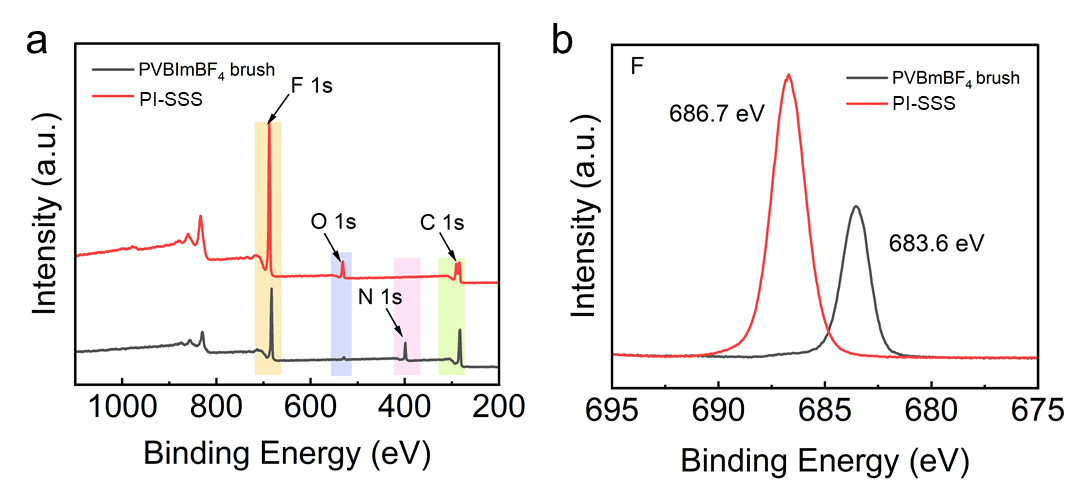


1. X-ray photoelectron spectroscopy full spectrum (a) and fine spectrum of F (b) of PVBImBF_4_ brush and PI-SSS.

The appearance stretching peaks have corresponded to C 1s (284.8 eV), O 1s (532.0 eV), F 1s (686.7 eV), respectively. In the interaction of the electron with the dipole, F moves to a higher binding energy (blueshift).


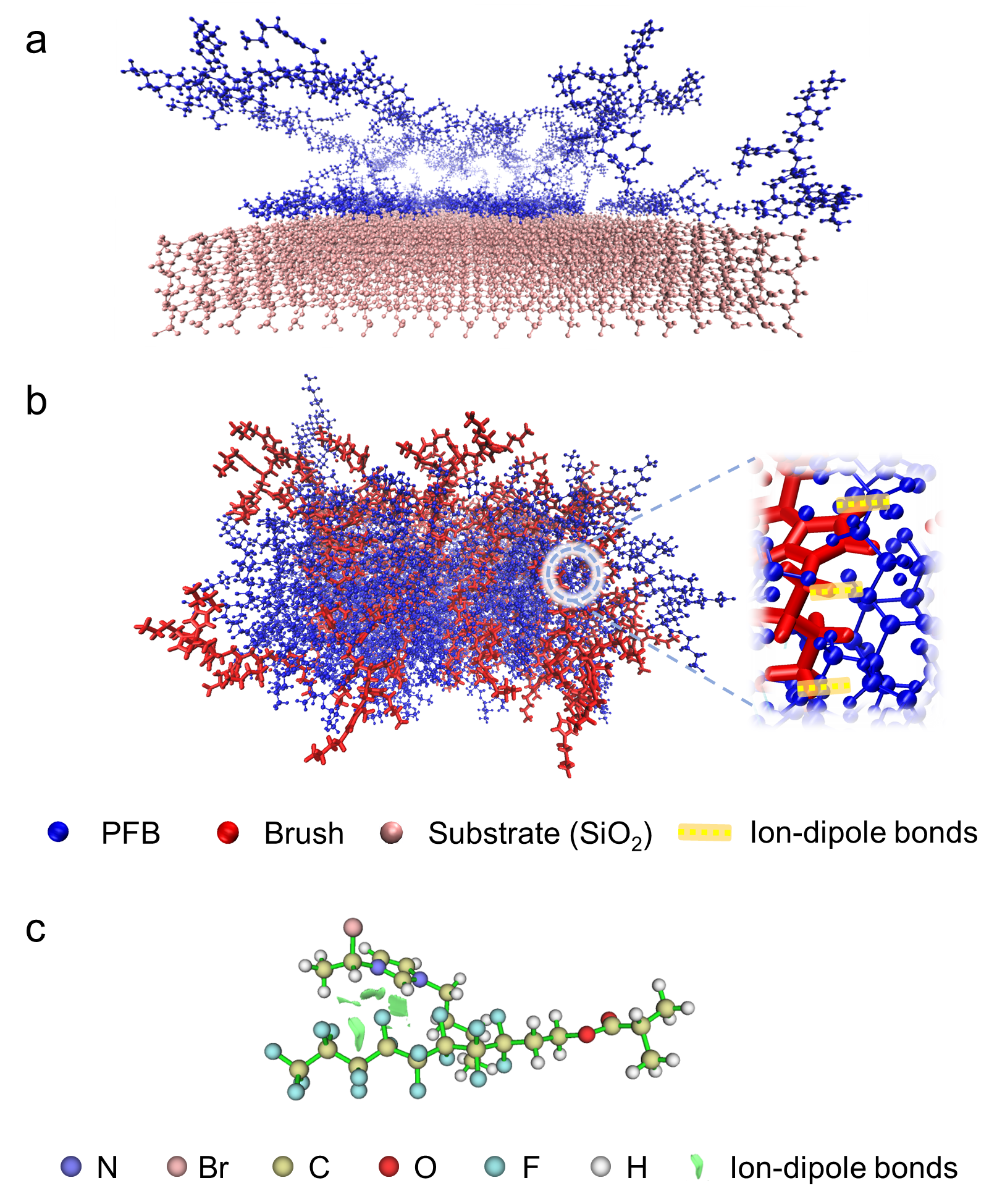


1. Molecular dynamics snapshot of substrate (a) and polymer brushes (b) adhesion with PFB copolymer. (c) Schematic diagram of density functional simulation of PFB and brush.

Molecular dynamics simulations initially revealed stronger binding forces between PFB and polymer brush-functionalized surfaces compared to the bare substrate (SiO_2_), suggesting improved interfacial adhesion. These observations were further corroborated by DFT, which derived absolute binding energies of ion-dipole bonds (-0.532 eV) to confirm the stabilizing role of the polymer brushes. See Sections 1.6.5 and 1.6.6 for the specific simulation process.


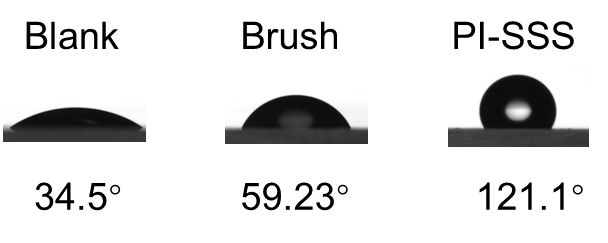


1. Contact angles of the liquids (4 μL) with water on different surfaces, including blank glass, brush-coated glass, and PI-SSS-coated glass.


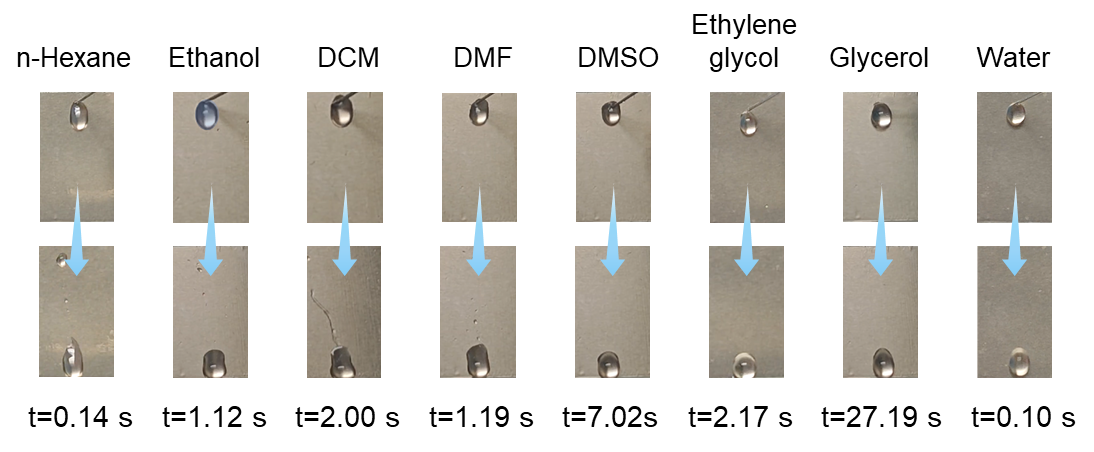


1. The sliding behavior tests of different surface tension liquids on PI-SSS (with a droplet of 20 μL and tilted angle of 30°). (Liquids include n-Hexane (17.9 mN·m^-1^), Ethanol (22.3 mN·m^-1^), DCM (27.2 mN·m^-1^), DMF (37.1 mN·m^-1^), DMSO (43.5 mN·m^-1^), Ethylene glycol (46.7 mN·m^-1^), Glycerol (61.9 mN·m^-1^), and water (72.8 mN·m^-1^).)


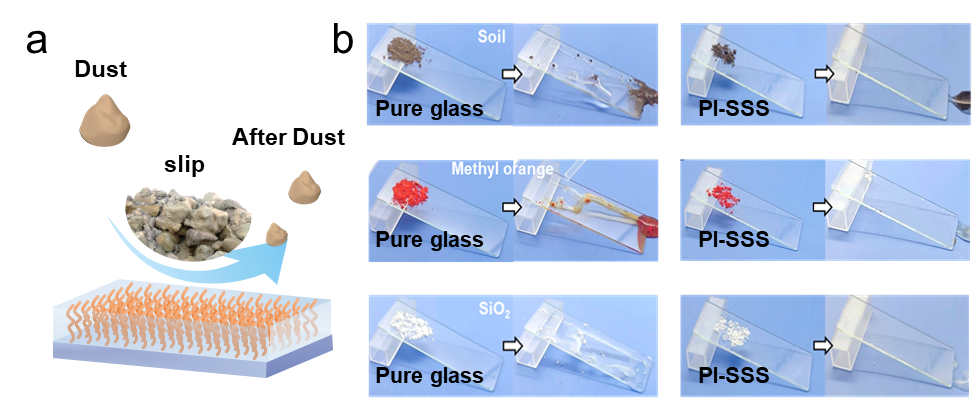


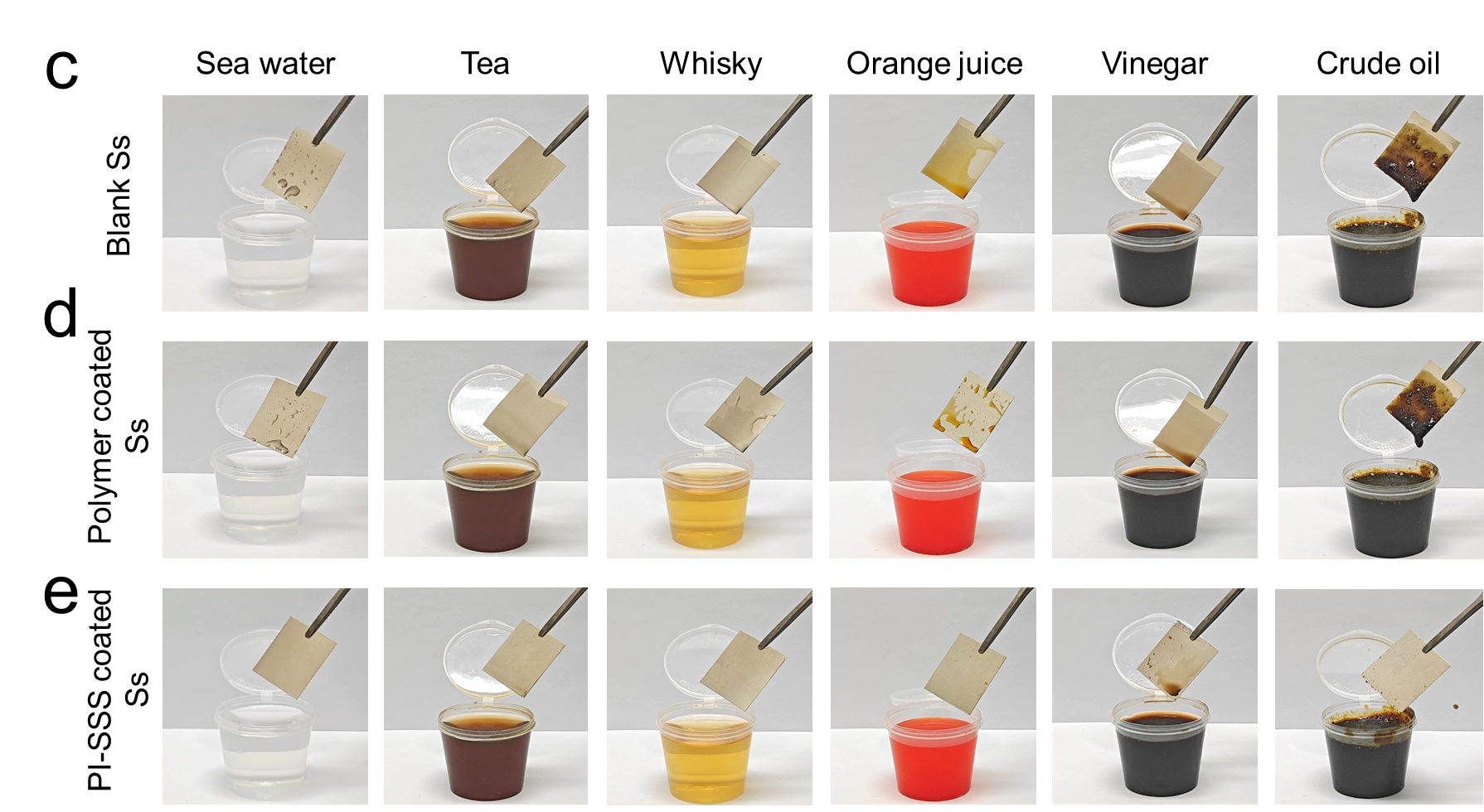


1. Self-cleaning performance of PI-SSS against solid model contaminants, including soil, methyl orange, and SiO_2_. Optical images of blank 316L SS (a), brushes coated 316L SS (d), and PI-SSS coated 316L SS (e) after contamination with liquid contaminants (sea water, tea, whisky, orange juice, vinegar, and crude oil).


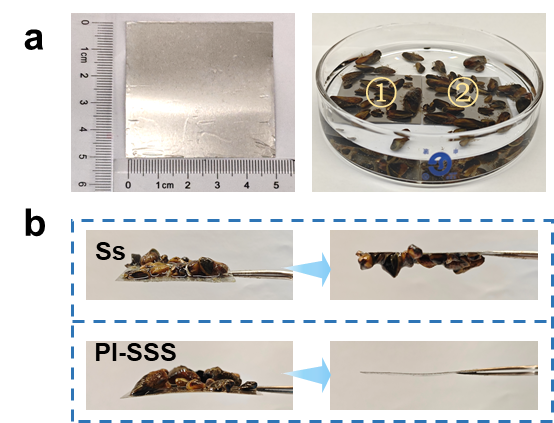


1. Anti-mussel adhesion tests on blank 316L SS and PI-SSS-coated 316L SS.

Mussels, crustacean marine organisms, are widely distributed in coastal and offshore areas. They are capable of secreting mucus that adheres to the surfaces of various types of equipment used in waterborne operations, thus seriously hampering normal operation^[9]^. To verify the antifouling ability of the PI-SSS, we tested it in a mussel growth solution. The results showed that mussels had difficulty adhering to the surface of PI-SSS, whereas slime was produced and firmly adsorbed in blank 316L SS.


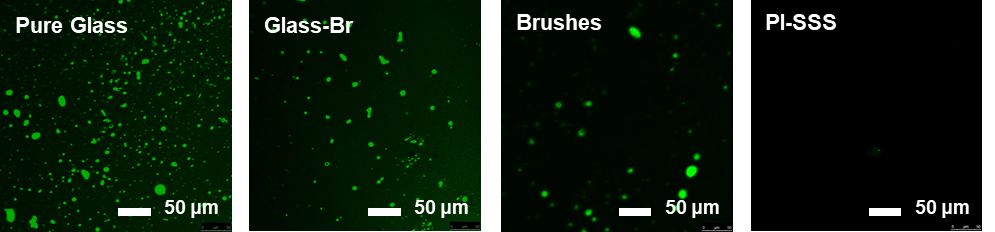


1. The fluorescence microscope photos of anti-protein adhesion tests on blank SiO_2_ surface, SiO_2_-Br, PVBImBF_4_ brush, and PI-SSS.


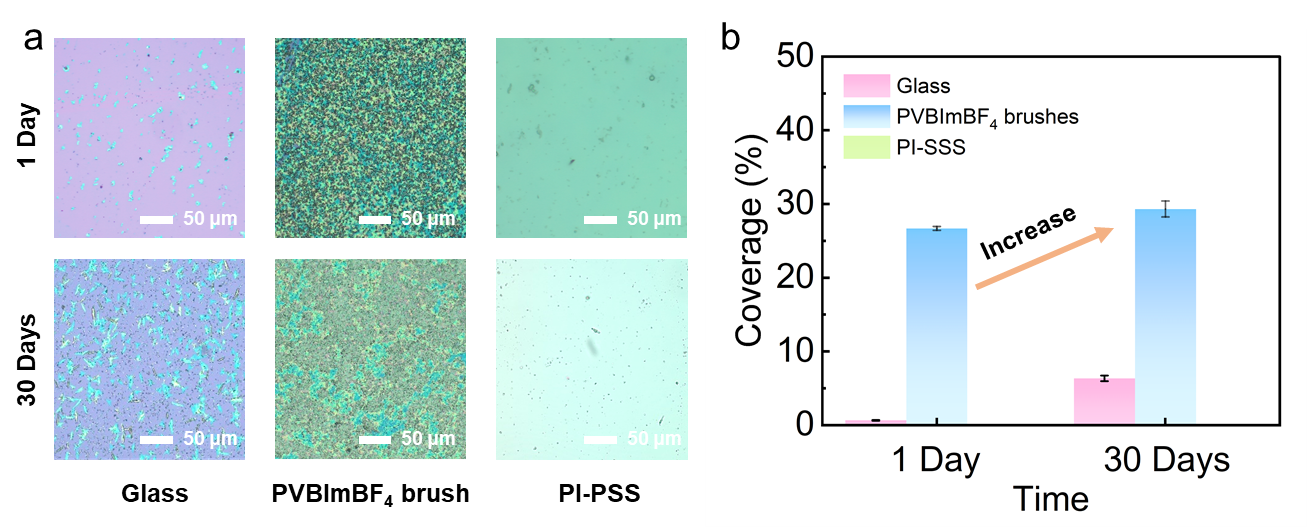


1. Algae resistance of PI-SSS. (a) Fluorescence microscope photos of anti-P. *tricornutum* adhesion tests from 1 to 30 days. (b) Statistical results for the algal coverage of various substrates (pure glass, PVBImBF_4_ brushes-coated glass, and PI-SSS-coated glass).

Chlorella, a model organism representing marine algal bio-pollutants, was selected for the assessment of the persistence of PI-SSS on marine antifouling behavior over a period of up to one month^[4]^. Samples were immersed in suspension for 1 and 30 days and photographed with an optical microscope.


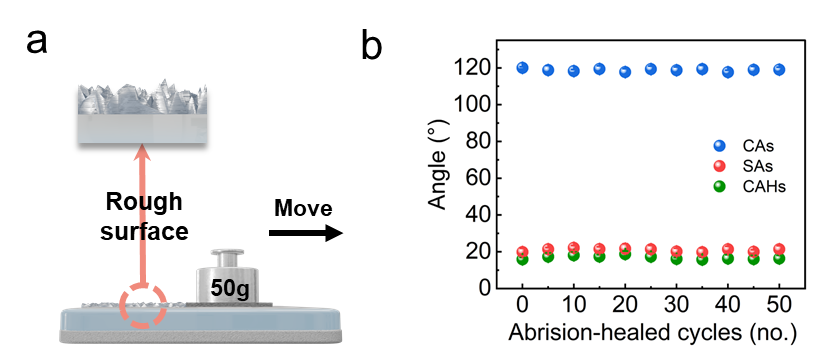


1. (a) Schematic illustration of the adhesion/peeling cycles. (b) Variation in CAs, SAs, and CAHs of PI-SSS with sandpaper friction.


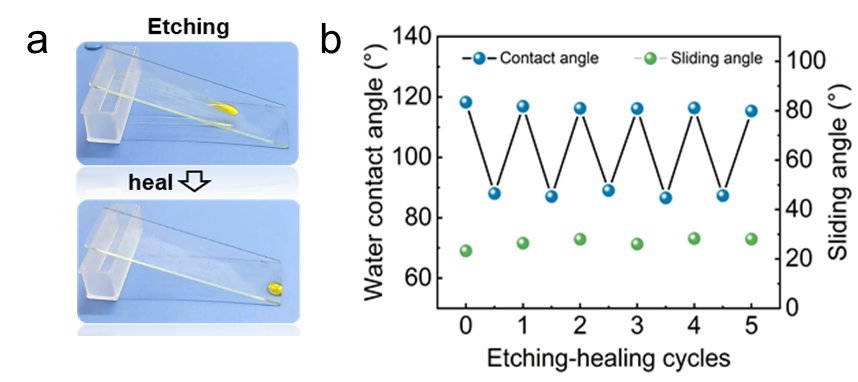


1. (a) Images show the slide after being etched with PI-SSS and heat-assisted repair.


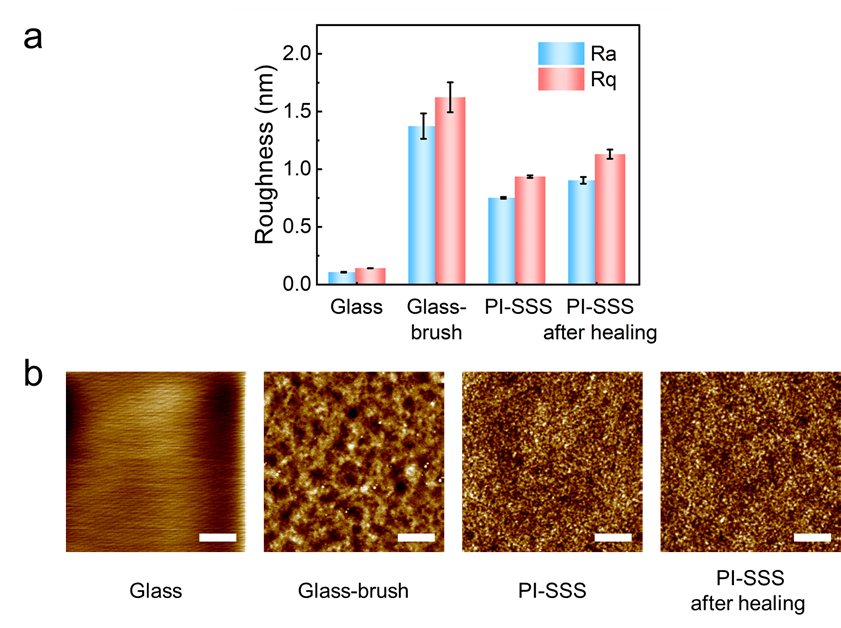


1. Surface roughness parameters, namely, average roughness (Ra) and root-mean-square roughness (Rq) (a) and SPM topography images (b) of blank glass, polymer brush, PI-SSS, and PI-SSS after self-healing (bar scale: 1 μm)


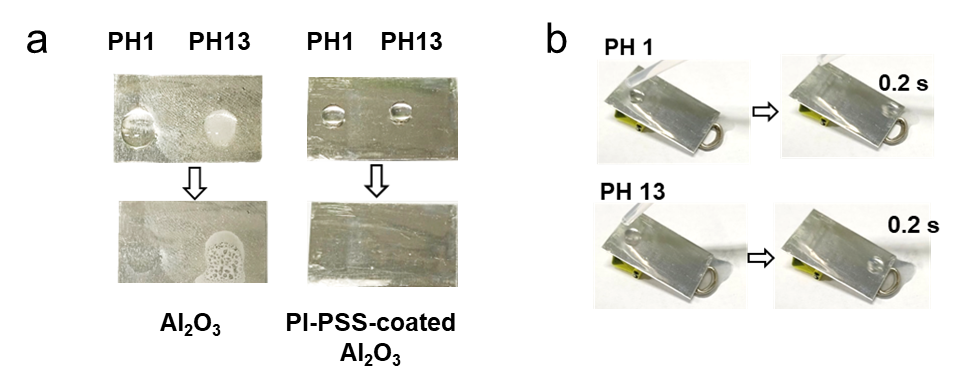


1. Optical images (a) and sliding behavior (b) of the blank Al_2_O_3_ surface and the PI-SSS-coated Al_2_O_3_ surface after acid-base corrosion.


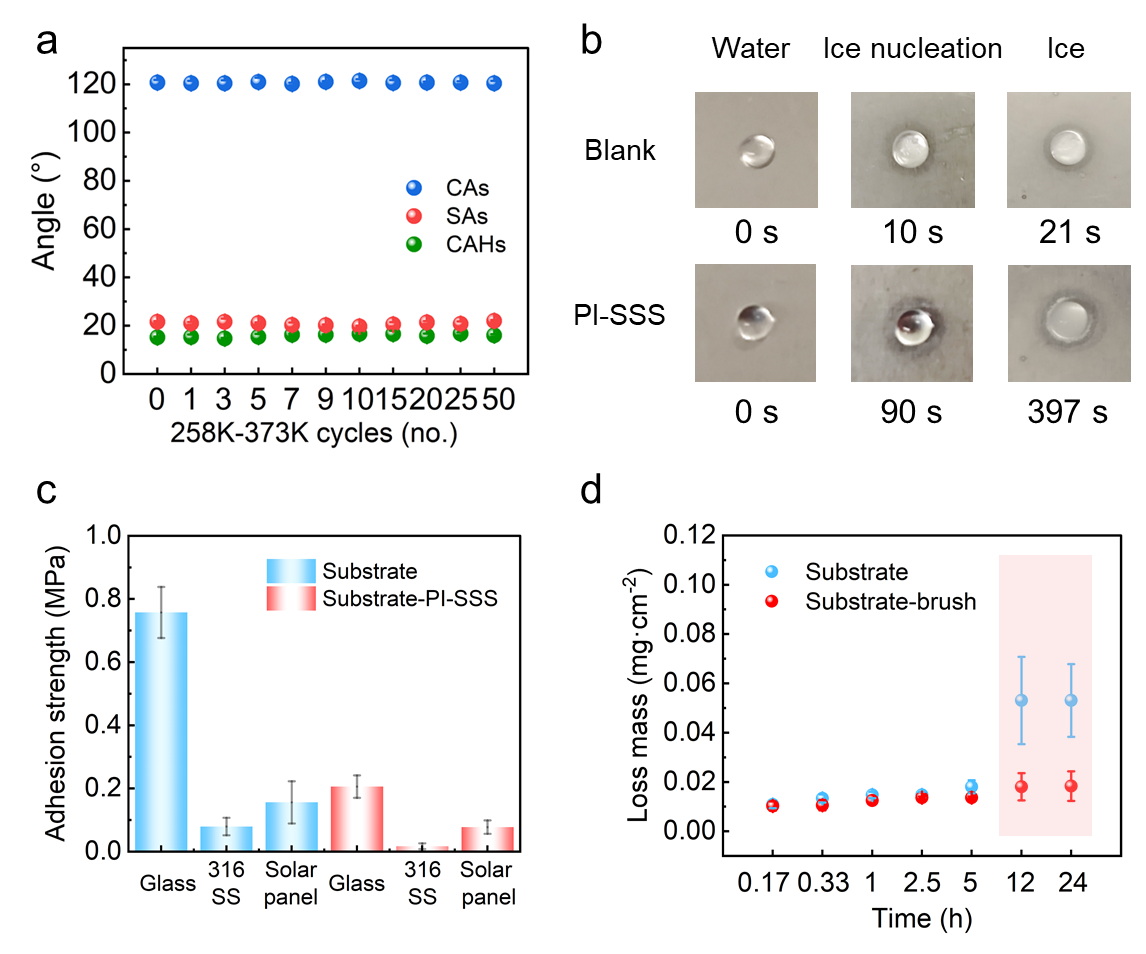


1. (a) Variation in CAs, SAs, and CAHs of PI-SSS with cyclic high-low temperature (-15°C to 100°C, 50 cycles). (b) The freezing of water on its surface. (c) Ice adhesion strength on various substrates, including blank glass, blank 316 SS, blank solar panel, PI-SSS-coated glass, PI-SSS-coated 316 SS, and PI-SSS-coated solar panel. (d) Mass loss of PFB from blank and polymer brush-coated 316 SS substrates held vertically (θ = 90°) at 100 °C.

This coating maintained excellent thermal cycling stability, as evidenced by minimal variations in water contact angle and contact angle hysteresis angle after 50 accelerated aging cycles between -15°C and 100°C (Figure S19a). As demonstrated in Figure S19, a 20 μL water droplet froze within 10 s on the bare surface at -15°C. In contrast, the PI-SSS-coated surface significantly delayed ice formation, with droplet crystallization initiating at 90 s and complete freezing occurring only after 397 s, demonstrating remarkable anti-icing capability (Figure S19b). Furthermore, PI-SSS coatings significantly reduced ice adhesion on glass (0.206 MPa), 316 SS (0.0168 MPa), and solar panel (0.0772 MPa)substrates (Figure S19c). Meanwhile, the brush-modified 316 SS retained most of the lubricant (0.018 ± 0.055 mg·cm^-2^), demonstrating the polymer brush’s role in stabilizing and anchoring the lubricant layer against detachment (Figure S19d).


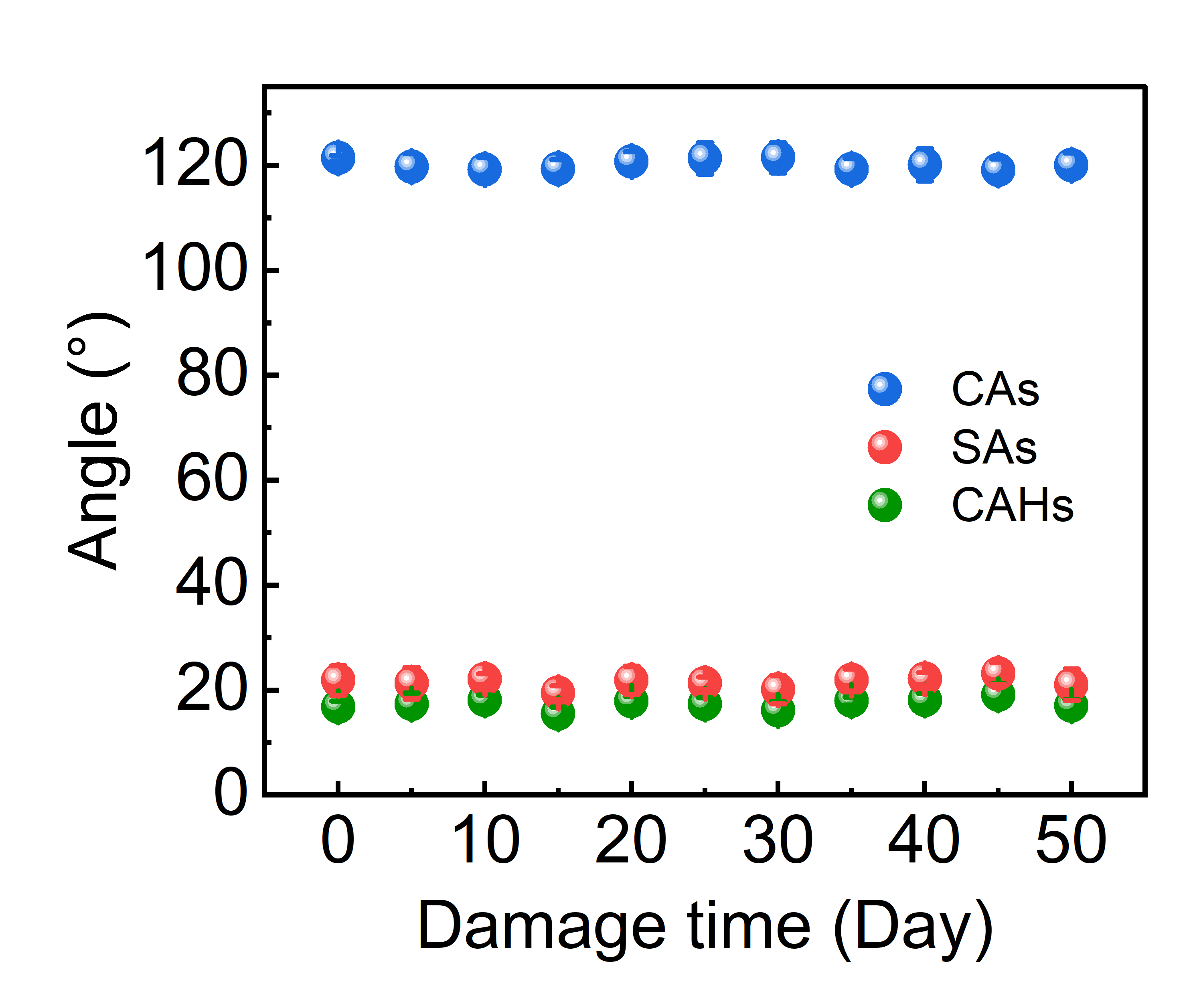


1. Variation in CAs, SAs, and CAHs of PI-SSS with soaking in seawater for 50 days.


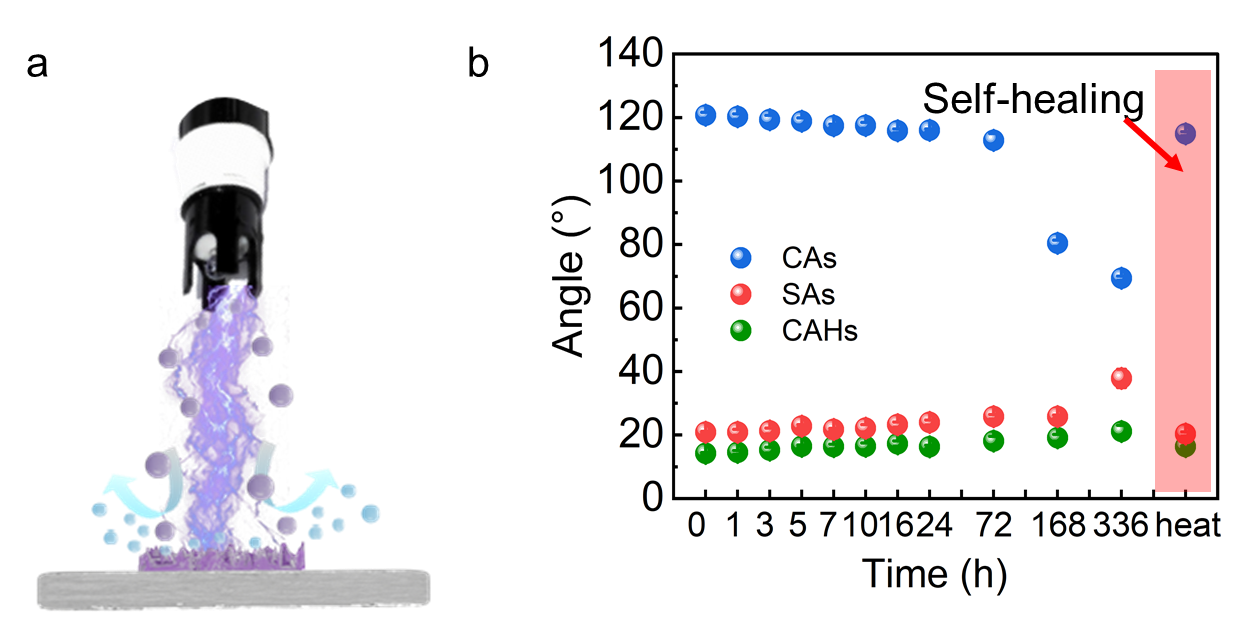


1. Variation in CAs, SAs, and CAHs of PI-SSS with ultraviolet destruction.

Under continuous UV exposure (365 nm), the PI-SSS coating exhibited significant degradation after 7 days, as evidenced by measurable changes in water sliding angle and contact angle. However, thermal treatment at 100°C for 2 min completely restored the surface properties to their initial state (SAs= 20°±1.5°, CAs=120°±2.0°). This recovery phenomenon suggests that UV irradiation likely cleaves the cross-linked networks of PFB polymer chains, while subsequent heating enables molecular rearrangement through liquid-phase chain interdiffusion.


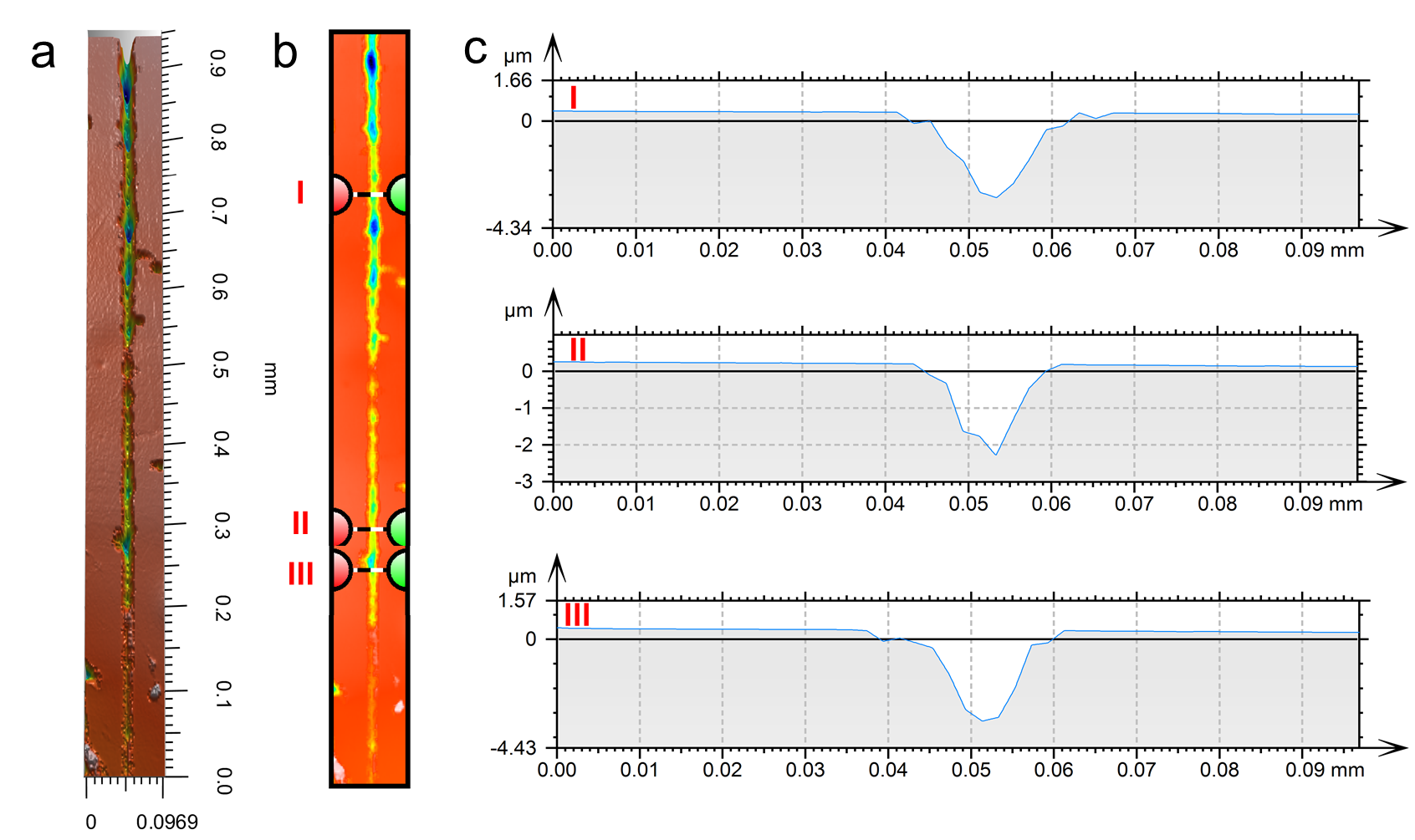


1. Three-dimensional topographic image (a) and two-dimensional surface view (b) of the knife scratch on the PI-SSS coating. (c) Cross-sectional profiles were measured at three positions along the scratch.


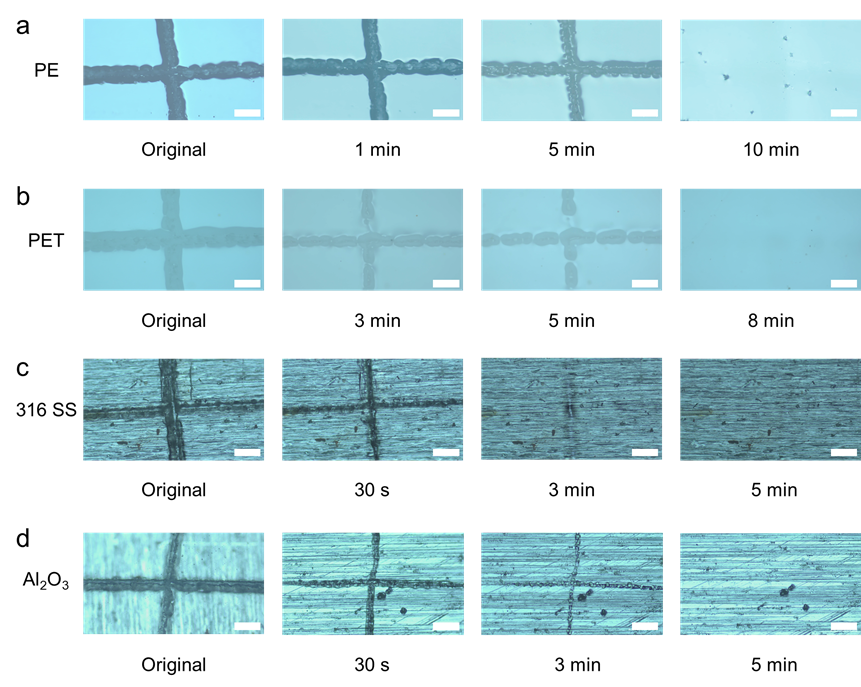


1. Microscopic images of PI-SSS showing thermally assisted healing of physical damage on different substrates, including PE (a), PET (b), 316 SS (c), and Al_2_O_3_ (d) (Bar scale: 200 μm).


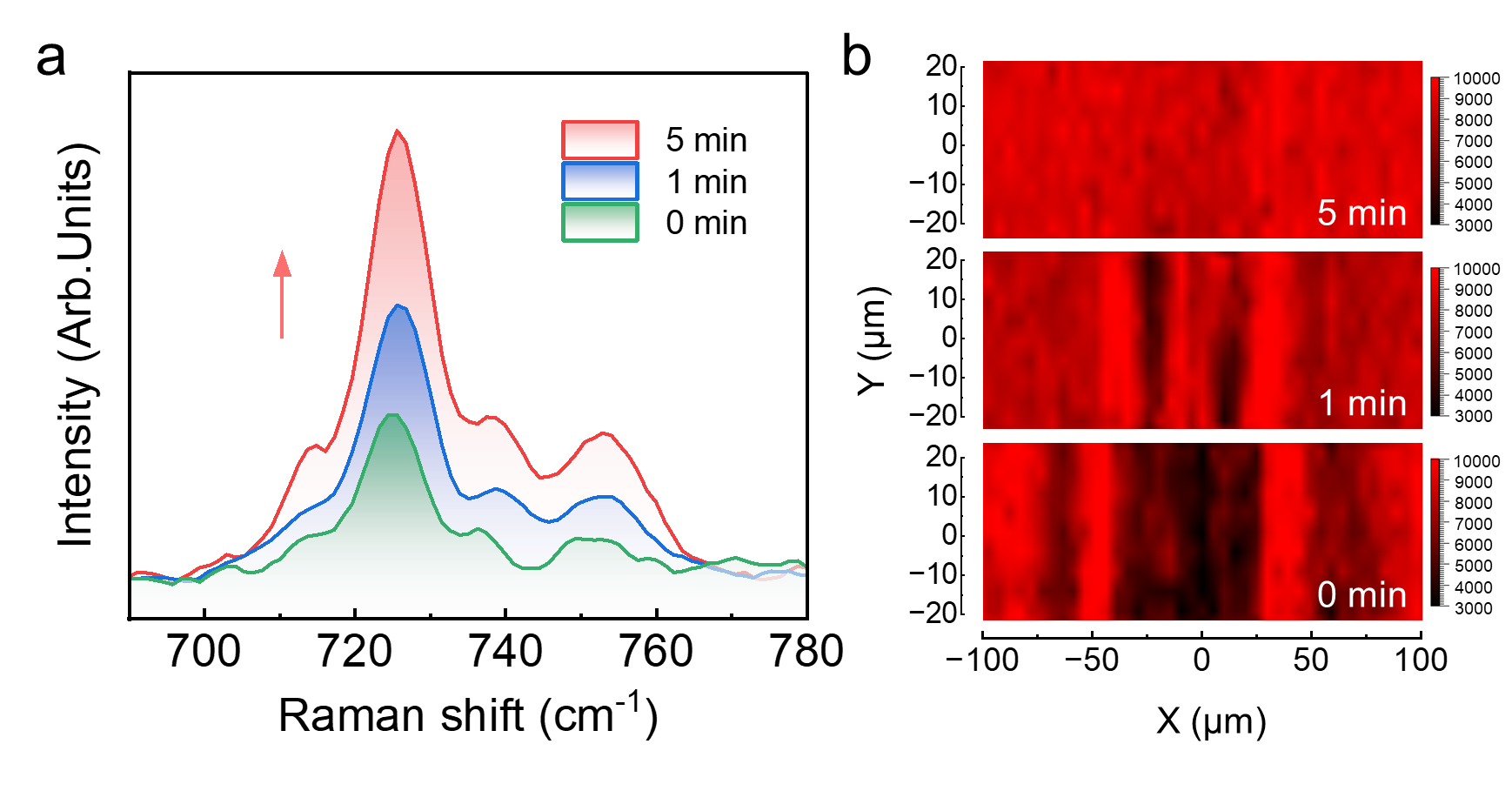


1. Raman spectral evolution of PI-SSS during the healing process.


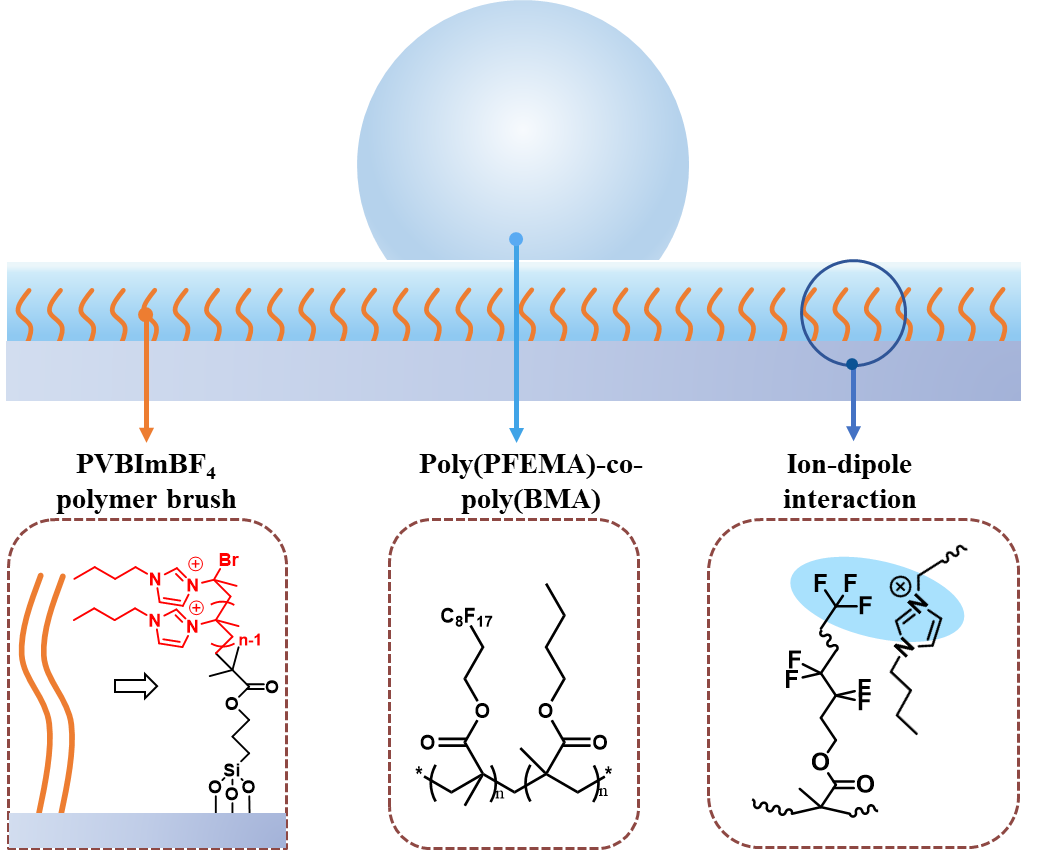


1. Fabrication of PI-SSS via dynamic ion-dipole interactions between surface-grafted polymer chains and lubricating molecules.

To construct a plant cuticle-inspired solid slippery surface (PI-SSS) with multifunctional properties, we selected key materials VBImBF₄ and PFB based on their synergistic interactions and performance. The ionic monomer VBImBF₄ was polymerized via SI-Cu⁰CRP to form stable PVBImBF₄ polymer brushes containing imidazolium groups. PFB possesses low surface energy and abundant dipolar fluorine groups, enabling it to form strong ion–dipole interactions with the imidazolium moieties of PVBImBF₄ brushes.

1. **Supplementary Movies**

**Movie S1.** Bouncing behavior of different liquids on the surface of PI-SSS-coated glass.

1. **References**

[1] J. Yan, B. Li, B. Yu, W. T. S. Huck, W. Liu, F. Zhou, "Controlled Polymer-Brush Growth from Microliter Volumes using Sacrificial-Anode Atom-Transfer Radical Polymerization" *Angew. Chem. Int. Ed.* **2013**, *52*, 9125-9129.

[2] T. Zhang, Y. Du, F. Müller, I. Amin, R. Jordan, "Surface-initiated Cu(0) mediated controlled radical polymerization (SI-CuCRP) using a copper plate" *Poly. Chem.* **2015**, *6*, 2726-2733.

[3] W. Niu, G. Y. Chen, H. Xu, X. Liu, J. Sun, "Highly Transparent and Self-Healable Solar Thermal Anti-/Deicing Surfaces: When Ultrathin MXene Multilayers Marry a Solid Slippery Self-Cleaning Coating" *Adv. Mater.* **2022**, *34*, 2108232.

[4] C. Yang, X. Ding, R. J. Ono, H. Lee, L. Y. Hsu, Y. W. Tong, J. Hedrick, Y. Y. Yang, "Brush-Like Polycarbonates Containing Dopamine, Cations, and PEG Providing a Broad-Spectrum, Antibacterial, and Antifouling Surface via One-Step Coating" *Adv. Mater.* **2014**, *26*, 7346-7351.

[5] M. J. Abraham, T. Murtola, R. Schulz, S. Páll, J. C. Smith, B. Hess, E. Lindahl, "GROMACS: High performance molecular simulations through multi-level parallelism from laptops to supercomputers" *SoftwareX* **2015**, *1*, 19-25.

[6] W. Humphrey, A. Dalke, K. Schulten, "VMD: visual molecular dynamics" *J. Mol. Graphics* **1996**, *14*, 33-38.

[7] T. Lu, F. Chen, *J. Comput. Chem.* "Multiwfn: A multifunctional wavefunction analyzer" **2012**, *33*, 580-592.

[8] T. Lu, Q. Chen, "Visualization analysis of weak interactions in chemical systems" *Compr. Comput. Chem.* **2024**, *2*, 240-264.

[9] S. Amini, S. Kolle, L. Petrone, O. Ahanotu, S. Sunny, C. N. Sutanto, S. Hoon, L. Cohen, J. C. Weaver, J. Aizenberg, N. Vogel, A. Miserez, "Preventing mussel adhesion using lubricant-infused materials" *Science* **2017**, *357*, 668-673.
